# Development of novel NEMO-binding domain mimetics for inhibiting IKK/NF-κB activation

**DOI:** 10.1371/journal.pbio.2004663

**Published:** 2018-06-11

**Authors:** Jing Zhao, Lei Zhang, Xiaodong Mu, Christelle Doebelin, William Nguyen, Callen Wallace, Daniel P. Reay, Sara J. McGowan, Lana Corbo, Paula R. Clemens, Gabriela Mustata Wilson, Simon C. Watkins, Laura A. Solt, Michael D. Cameron, Johnny Huard, Laura J. Niedernhofer, Theodore M. Kamenecka, Paul D. Robbins

**Affiliations:** 1 Department of Molecular Medicine and the TSRI Center on Aging, The Scripps Research Institute, Jupiter, Florida, United States of America; 2 Department of Immunology, University of Pittsburgh School of Medicine, Pittsburgh, Pennsylvania, United States of America; 3 Department of Orthopaedic Surgery, McGovern Medical School, University of Texas Health Science Center at Houston, Houston, Texas, United States of America; 4 Department of Cell Biology, University of Pittsburgh School of Medicine, Pittsburgh, Pennsylvania, United States of America; 5 Department of Neurology, University of Pittsburgh, Pennsylvania, United States of America; 6 Department of Health Informatics and Information Management, College of Nursing and Health Professions, University of Southern Indiana, Evansville, Indiana, United States of America; 7 Department of Immunology and Microbiology, The Scripps Research Institute, Jupiter, Florida, United States of America; Stanford University, United States of America

## Abstract

Nuclear factor κB (NF-κB) is a transcription factor important for regulating innate and adaptive immunity, cellular proliferation, apoptosis, and senescence. Dysregulation of NF-κB and its upstream regulator IκB kinase (IKK) contributes to the pathogenesis of multiple inflammatory and degenerative diseases as well as cancer. An 11–amino acid peptide containing the NF-κB essential modulator (NEMO)-binding domain (NBD) derived from the C-terminus of β subunit of IKK, functions as a highly selective inhibitor of the IKK complex by disrupting the association of IKKβ and the IKKγ subunit NEMO. A structure-based pharmacophore model was developed to identify NBD mimetics by in silico screening. Two optimized lead NBD mimetics, SR12343 and SR12460, inhibited tumor necrosis factor α (TNF-α)- and lipopolysaccharide (LPS)-induced NF-κB activation by blocking the interaction between IKKβ and NEMO and suppressed LPS-induced acute pulmonary inflammation in mice. Chronic treatment of a mouse model of Duchenne muscular dystrophy (DMD) with SR12343 and SR12460 attenuated inflammatory infiltration, necrosis and muscle degeneration, demonstrating that these small-molecule NBD mimetics are potential therapeutics for inflammatory and degenerative diseases.

## Author summary

Aberrant up-regulation of the transcription factor nuclear factor κB (NF-κB) and the IκB kinase (IKK) that regulates NF-κB is associated with a variety of inflammatory and degenerative diseases in humans, including aging. Thus, development of effective and specific drugs able to decrease IKK/NF-κB activity has significant therapeutic potential. In this study, a structure-derived computational approach was used to screen for small-molecule inhibitors of the protein–protein interaction between the IKKß and IKKγ subunits of the IKK complex. We identified and developed a novel class of small molecules that selectively inhibit IKK/NF-κB activation by dissociating the IKK complex without affecting c-Jun N-terminal kinase (JNK)/p38-mitogen-activated protein kinase (MAPK) signaling. These novel molecules reduce lipopolysaccharide (LPS)-induced acute inflammation in mice and improve muscle pathology in the *mdx* mouse model of Duchenne muscular dystrophy (DMD), suggesting that they would have potential clinical utility.

## Introduction

Nuclear factor κB (NF-κB) is a transcription factor essential for regulating immune responses, cell proliferation, apoptosis, embryonic development, senescence, and cancer [1]. In mammalian cells, the NF-κB family is composed of 5 subunits, RelA/p65, RelB, C-Rel, p50 (p105/NF-κB1), and p52 (p100/NF-κB2), all containing a Rel-homology domain (RHD) required for homo- or heterodimerization [1]. NF-κB dimers are sequestered in the cytoplasm by an inhibitory protein IκBα, which masks the conserved nuclear localization sequence (NLS) of RelA/p65 to prevent nuclear translocation [1]. Upon stimulation, IκBα undergoes phosphorylation, polyubiquitination, and proteasome-mediated degradation, releasing the NF-κB dimers to enable nuclear translocation [1]. This activated NF-κB down- or up-regulates target gene expression by binding to the κB enhancer or promoter elements [1]. Inducers of NF-κB activity include pro-inflammatory cytokines, such as tumor necrosis factor α (TNF-α), interleukin-1 (IL-1), and lipopolysaccharide (LPS), as well as T-cell receptor (TCR) ligands, genotoxic, and oxidative stress [1].

NF-κB activation is regulated by the IκB kinase (IKK) complex, composed of 2 catalytic subunits, IKKα and IKKβ, and a regulatory subunit NF-κB essential modulator (NEMO)/IKKγ [2–4]. Domains in the C-termini of IKKα and IKKβ, required for interaction with the α-helical region in the N-terminus of NEMO, are termed NEMO-binding domains (NBDs) [5]. An 11–amino acid peptide derived from the NBD domain of IKKβ (amino acids 735–745) can disrupt the association of IKKβ and NEMO and reduce NF-κB activation when fused to a protein transduction domain (PTD) for intracellular delivery [5].

The NBD peptide has strong therapeutic effects in numerous inflammatory and degenerative disease models in mice and other species. Chronic, systemic administration of the NBD peptide attenuates macrophage-mediated muscle necrosis and degeneration in *mdx* mice, a murine model of Duchenne muscular dystrophy (DMD) as well as in the golden retriever muscular dystrophy (GRMD) canine model of DMD [6–8]. Similarly, the NBD peptide ameliorates active chronic colitis in IL-10-deficient mice without affecting NF-κB basal activity when administered systemically [9]. Intra-articular injection of NBD peptide also attenuates synovial inflammation and the severity of arthritis in a rat model of adjuvant arthritis [10]. It also ameliorates inflammation-induced osteoclastogenesis and arthritis by down-regulating NF-κB target genes, TNF-α, and IL-1β [11]. Systemic delivery of the NBD peptide reduces the severity of Parkinson’s disease by suppressing nigral microglial activation and reducing dopaminergic neuronal loss as well as alleviates nephropathy and atherosclerosis in type 1 diabetic mice [12–14]. In addition, the peptide prevents LPS-induced pulmonary inflammation in sheep and improves pulmonary function in a piglet model of acute respiratory distress syndrome by topical administration [15, 16]. Moreover, clinical testing of the NBD peptide for local treatment of canine diffuse large B cell lymphoma revealed a reduction in the proliferation of malignant B cells [17]. In addition, chronic systemic administration of NBD peptide delays the onset and reduces the severity of multiple aging symptoms and pathology in *Ercc1*^*-/Δ*^ mice, a mouse model of human progeria [18].

Despite these strong and varied therapeutic effects of PTD-NBD peptides in animal models, the expense of peptide synthesis, the short half-life of the peptide, and its lack of oral bioavailability limit its clinical use. Thus, development of small molecules that mimic the NBD peptide, targeting the NBD of IKKβ to disrupt its binding to NEMO, would have clinical utility. Here, a structure-based pharmacophore model that mimics these interactions was derived from the crystal structure of the IKK complex, followed by virtual screening using this model against commercially available databases of drug-like molecules. The resulting hits were prioritized using in silico Absorption, Distribution, Metabolism, Excretion, and Toxicity (ADME/Tox) filtering and molecular docking to determine the higher-affinity hits. Using these as starting points, multiple rounds of medicinal chemistry optimization resulted in the discovery of compounds capable of inhibiting LPS- and TNF-α-induced NF-κB activation by disrupting the association between IKKβ and NEMO. Also, these compounds exhibited potent therapeutic effects in murine models of LPS-induced endotoxemia and DMD, suggesting their potential as therapeutic drugs for clinical management of diseases driven by IKK/NF-κB activation.

## Results

### Generation of a structure-based pharmacophore model using a computational approach based on the conserved interactions between IKKβ and NEMO

**Fig 1:**
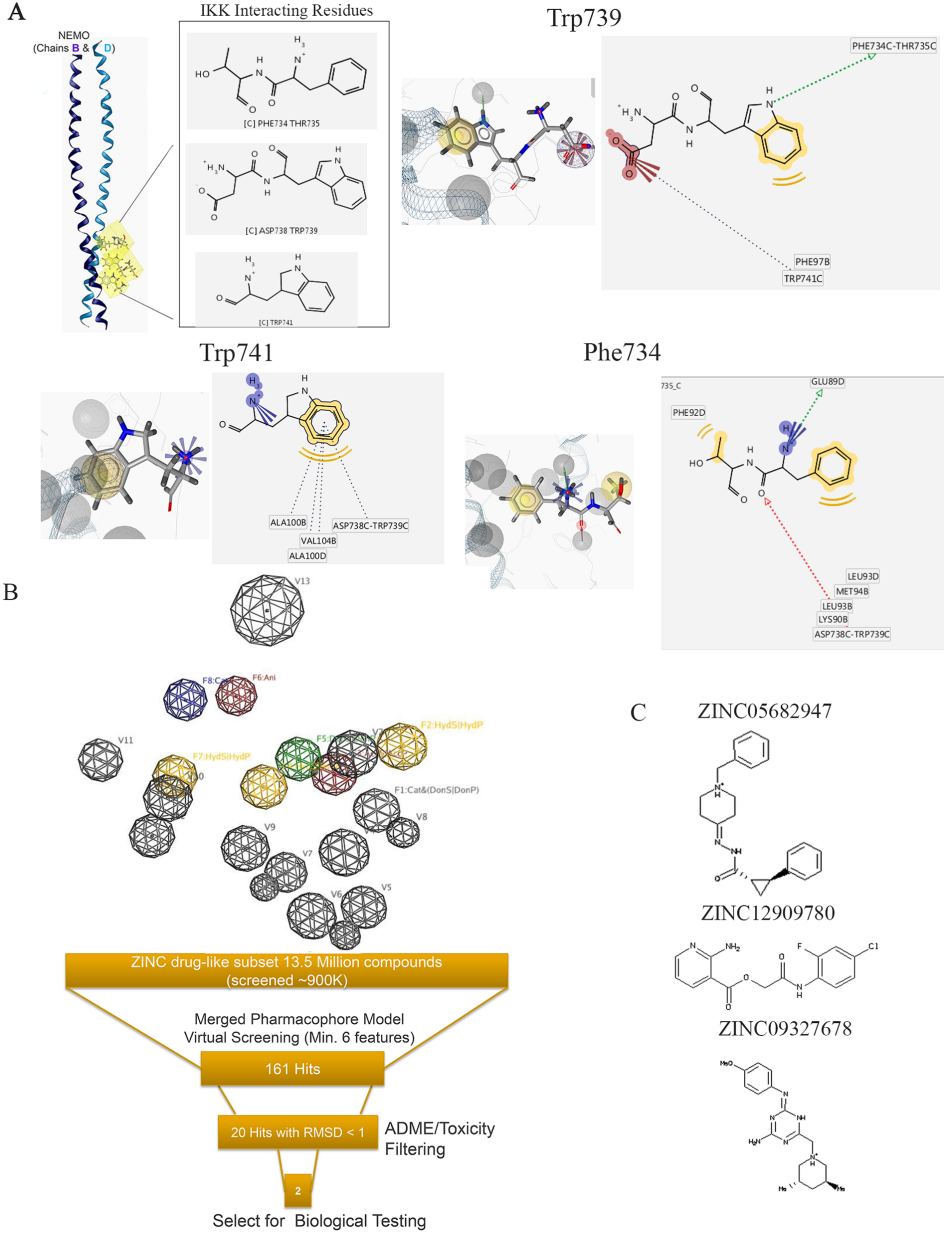
The development of a structure-based pharmacophore model for in silico screen of NBD mimetics. (A) Interacting residues extracted from the X-ray structure of the NEMO/IKKβ complex (PDB ID: 3BRV) used in the generation of the structure-based pharmacophore model [21]. (B) A 3D representation of the structure-based pharmacophore model. Three hydrophobic groups (F2, F4, and F7: yellow spheres), 1 hydrogen-bond acceptor features (F3: red sphere), 1 hydrogen-bond donor (F5: green sphere), 1 positive ionizable area (F8: blue sphere), 1 negative ionizable (F6: red sphere), and 13 excluded volumes (gray spheres) are shown. (C) Chemical structures of 3 compounds that entered biological testing [24]. 3D, three-dimensional; IKK, IκB kinase; NBD, NEMO-binding domain; NEMO, NF-κB essential modulator; PDB, Protein Data Bank.

Recognition of small molecules by proteins is largely mediated by molecular surface complementarities [19, 20]. Thus, the site of protein–protein interaction between NEMO and IKKβ potentially is a good target for in silico drug screening. To investigate the chemical features essential in the protein–protein interaction, the X-ray structure of the NEMO/IKKβ complex retrieved from the Protein Data Bank (PDB) (ID 3BRV) was used to generate a structure-based pharmacophore (Fig 1A) [21] using the pharmacophore generation module of LigandScout [22, 23]. Each interacting atom from each residue was “translated” into a pharmacophoric feature, resulting in the structure-based pharmacophore (Fig 1B) consisting of 8 features and 13 exclusion volumes, representing important atoms from the protein’s environment.

This pharmacophore model was used to screen a subset of the drug-like ZINC 10.0 database set (approximately 13.5 million compounds) [24]. We identified 161 compounds that matched at least 6 features out of 8 of the pharmacophore model. Twenty hits had a root-mean-square deviation (RMSD) <1 and were further prioritized using ADME/Tox-predicted properties. Three compounds successfully passed these filters (Fig 1C).

### Identification of small-molecule inhibitors of NF-κB activation

**Fig 2:**
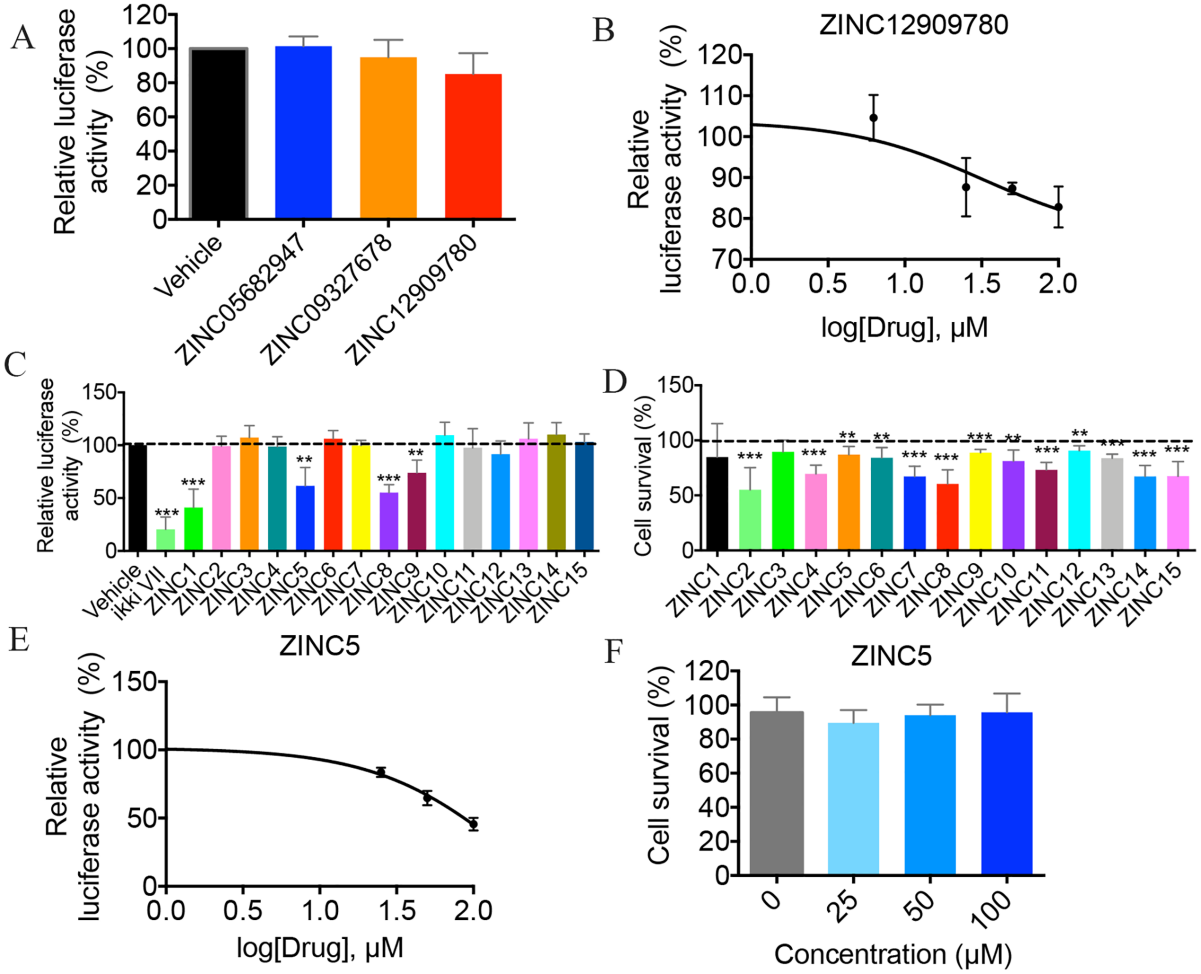
Identification of small molecules inhibiting TNF-α-induced NF-κB activation. (A) HEK293 cells stably expressing NF-κB luciferase reporter were pretreated with indicated small molecules at 100 μM for 30 min, followed by stimulation with TNF-α (10 ng/ml) for 3 h. Luciferase activity was normalized to untreated stimulated control cells. Data show the mean values of 2 independent experiments +/− SD. (B) The dose-dependent response of ZINC12909780 was tested by NF-κB luciferase assay in HEK293 cells. Three independent experiments were performed, and data shown represent mean +/− SD. (C) HEK293 cells were treated with derivatives of ZINC12909780 at 100 μM, and NF-κB luciferase assay was performed to screen for small molecules with greater NF-κB inhibitory effect. Data show the pooled results of 5 independent experiments and are the mean +/− SD. (D) HEK293 cells were cultured in the presence of 100 μM of indicated derivatives for 24 h, and cell survival was determined by MTT assay. Cell viability was calculated by normalizing to untreated cells. (E) Dose-dependent inhibitory effects were determined for ZINC5 at 0, 25, 50, and 100 μM by using NF-κB luciferase assay. (F) HEK293 were cultivated in the presence of ZINC5 at listed concentrations for 24 h, and cell survival was assessed by MTT assay. Data represent the mean +/− SD from 4–5 independent experiments. *P < 0.05; **P < 0.01; ***P < 0.001. Underlying data can be found in S1 Data. HEK293, human embryonic kidney 293 cells; MTT, 3-(4,5-dimethylthiazol-2-yl)-2,5-dephenyltetrazolium bromide colormetric assay for assessing cell metabolic activity; NF-κB, nuclear factor κB; TNF-α; tumor necrosis factor α; SD, standard deviation.

To determine whether the small molecules identified by the in silico screening inhibit NF-κB activation, a HEK293 cell line stably expressing a luciferase reporter driven by a synthetic, NF-κB-dependent promoter was utilized [25]. To induce NF-κB activation, cells were treated with 10 ng/mL of TNF-α and harvested 3 h post treatment for analysis of luciferase activity. Treatment with ZINC12909780 slightly down-regulated NF-κB activation at a concentration of 100 μM, whereas the luciferase activity remained unchanged in cells treated with ZINC05682974 or ZINC09327678 (Fig 2A). To determine whether ZINC12909780 inhibits NF-κB in a dose-dependent manner, concentrations were tested at 0, 6.25, 25, 50, and 100 μM. Only the high concentrations (50 and 100 μM) of the compound were able to inhibit TNF-α-induced NF-κB activation significantly (Fig 2B) [25].

To identify NBD mimetics with higher biological activity than ZINC12909780 or the NBD peptide, the ZINC 10.0 database (approximately 13.5 million compounds) was screened in silico for structurally similar compounds [26]. Fifteen analogs with a similarity score >90% were identified, and 13 that passed all the ADME/Tox filters were acquired for testing (S1 Table). Four of the compounds—ZINC9642366 (Zinc1), ZINC3369392 (Zinc5), ZINC3269261 (Zinc8), and ZINC3264658 (Zinc9), as well as an IKK active site inhibitor IKKi VII used as a positive control—lowered NF-κB activity robustly, while other analogs had minimal effects (Fig 2C). To rule out the possibility that the reduction observed in luciferase assays was due to drug toxicity, a colorimetric MTT assay using 3-(4,5-dimethylthiazol-2-yl)-2,5-dephenyltetrazolium bromide was performed to assess cell viability. Treatment with ZINC8 resulted in 40% of cell death at 24 h, suggesting that at least part of the reduction in luciferase activity could be attributed to cytotoxicity (Fig 2D). Because ZINC5 displayed potent NF-κB inhibitory efficacy in cell culture with little toxicity (less than 10%), it was tested for dose-dependent inhibition of NF-κB. ZINC5 showed a greater inhibitory effect compared to ZINC12909780 without overt cell toxicity (Fig 2E & 2F).

### NBD mimetics inhibit NF-κB DNA binding activity

**Fig 3:**
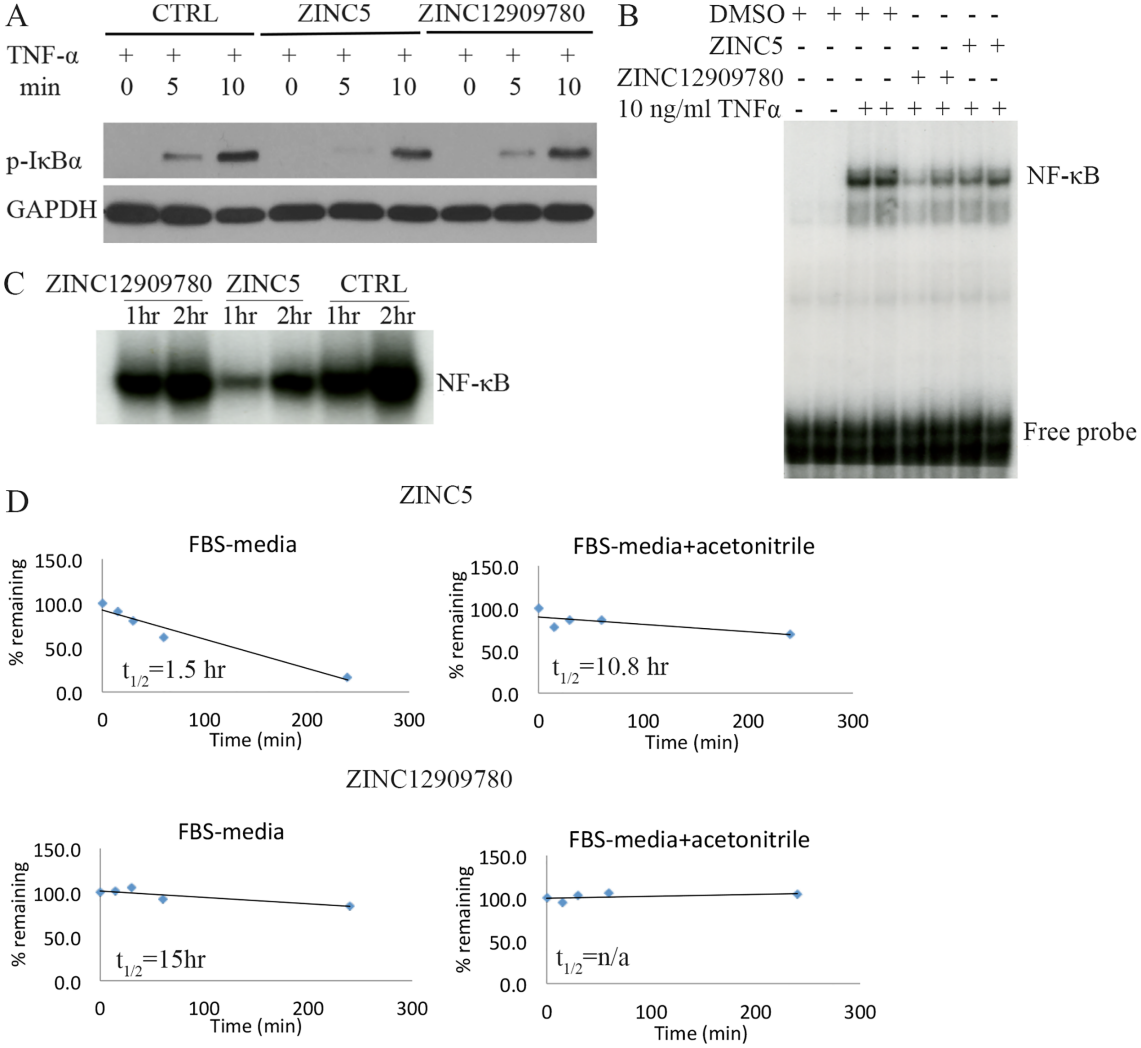
Two identified small molecules reduce NF-κB DNA binding activity in vivo and in vitro. (A) Levels of p-IκBα with or without treatment in response to TNF-α were determined by western blot analysis. HEK293 cells were pretreated with DMSO, ZINC12909780 (100 μM), and ZINC5 (100 μM) for 30 min followed by the stimulation of 10 ng/ml of TNF-α for 0, 5, and 10 min. Cell lysates were prepared for immunoblot against p-IκBα. GAPDH was used as a loading control. (B) EMSA analysis detecting NF-κB DNA binding activity was performed in C2C12 myoblasts. Cells were pretreated with 200 μM of DMSO, ZINC12909780, and ZINC5 in serum-free media for 1 h. TNF-α was then added to a final concentration of 10 ng/ml and incubated for 15 min. Nuclear extract was obtained for EMSA analysis. (C) EMSA analysis assessing NF-κB DNA binding activity in vivo was performed with quadriceps from mdx mice. Single-dose ZINC12909780 and ZINC5 at 10 mg/kg was given by i.p. Quadriceps was harvested at 1 h and 2 h post injection for EMSA analysis. (D) The time-course pharmacokinetics of ZINC5 and ZINC12909780 in FBS media or FBS media containing acetonitrile. Underlying data can be found in S1 Data. EMSA, electrophoretic mobility shift assay; HEK293, human embryonic kidney 293 cells; i.p., intraperitoneal; NF-κB, nuclear factor κB; t_1/2_, half-life; TNF-α; tumor necrosis factor α.

To confirm that the NBD mimetics reduce IKK kinase activity, phosphorylation of IκBα by IKK in response to 10 ng/ml of TNF-α was measured by western blot at 0, 5, and 10 min after stimulation. ZINC5 reduced the level of p-IκBα following stimulation, while ZINC12909780 led to a less robust reduction (Fig 3A). To determine whether the mimetics also reduce NF-κB DNA binding activity, electrophoretic mobility shift assay (EMSA) was conducted both in vitro and in vivo. ZINC5 and ZINC12909780 were tested in C2C12 cells (a mouse myoblast cell line) at 200 μM and were shown to inhibit TNF-α-induced NF-κB DNA binding activity (Fig 3B). Similarly, a single intraperitoneal (i.p.) injection of either of these 2 small molecules at 10 mg/kg inhibited NF-κB DNA binding activity in quadriceps that is chronically up-regulated in *mdx* mice (Fig 3C). However, ZINC5 was extremely unstable even in cell culture media containing FBS, while ZINC12909780 was relatively more stable. The addition of acetonitrile increased the stability of both ZINC compounds, especially ZINC 5 (Fig 3D).

### Optimization of the NBD mimetics

**Fig 4:**
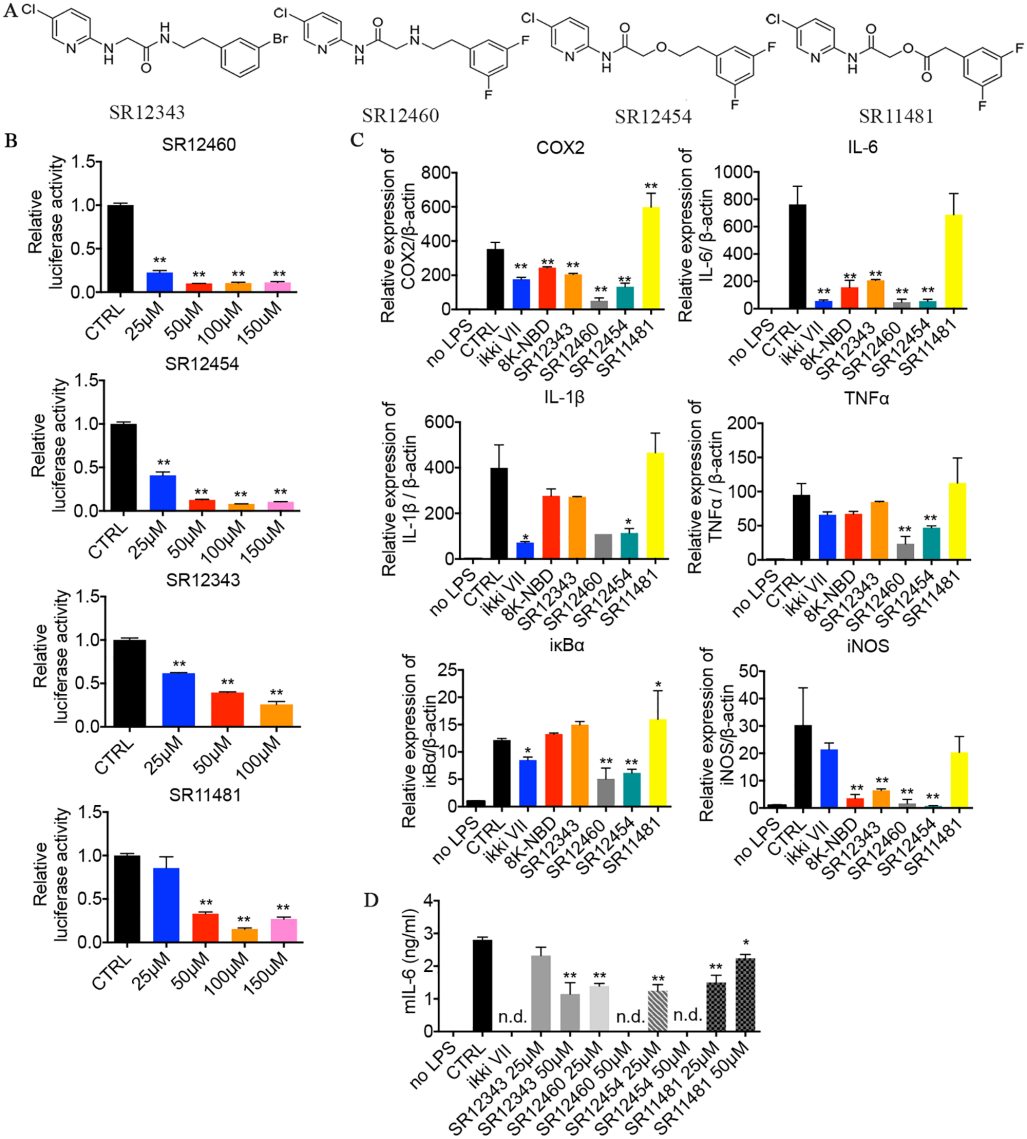
Modified lead NBD mimetics inhibit TNF-α- and LPS-induced NF-κB activation by disrupting the association between NEMO and IKKβ. (A) Structures of top NBD mimetics. (B) Measurement of NF-κB activation in response to TNF-α by using Dual-luciferase reporter assays. HEK293 cells cotransfected with NF-κB luciferase reporter and SV40-Renilla plasmids were pretreated with DMSO or listed small molecules at 0, 25, 50, 100, and 150 μM for 30 min, followed by the induction of TNF-α for 3 h. Data shown are representative of 2–3 independent experiments. (C) NBD mimetics down-regulated the expression of NF-κB target genes in response to LPS. Raw 264.7 cells were pretreated with indicated drugs for 30 min and then stimulated with 1 μg/ml of LPS for 2 h. Cells were then harvested for RNA extraction and qRT-PCR analysis. Drug concentrations used are as follows: IKKi VII (2 μM), 8K-NBD peptide (200 μM), SR12343 (50 μM), SR12460 (50 μM), SR12454 (50 μM), and SR11481 (50 μM). Data are representative of 2 independent experiments. (D) Mouse IL-6 production induced by LPS was down-regulated by NBD mimetics. Raw 264.7 cells pretreated with DMSO or drugs at indicated concentrations were exposed to 1 μg/ml of LPS for 24 h, and supernatant was collected for ELISA analysis of mouse IL-6. *P < 0.05; **P < 0.01. Underlying data can be found in S1 Data. HEK293, human embryonic kidney 293 cells; IKK, IκB kinase; IL-6, interleukin 6; LPS, lipopolysaccharide; NBD, NEMO-binding domain; n.d., nondetectable; NEMO, NF-κB essential modulator; NF-κB, nuclear factor κB; qRT-PCR, qualitative real-time polymerase chain reaction; TNF-α; tumor necrosis factor α.

ZINC5 and ZINC12909780 both contain ester bonds, leading to their rapid degradation in the presence of serum (Fig 3D). To improve bioactivity and stability, structure–activity relationship studies (SARs) were performed, and more than 100 small molecules were synthesized and tested. Four lead NBD mimetics that showed enhanced inhibitory effects, compared to the original ZINC compounds, were identified, including 3 non-esters—SR12343, SR12460, and SR12454—and 1 ester SR11481 (Fig 4A & 4B). These 3 non-esters significantly inhibited TNF-α-mediated NF-κB activation with half maximal inhibitory concentrations (IC_50_) of 11.34 μM, 20.24 μM, and 37.02 μM, respectively (Table 1, Fig 4B & S1A Fig). The ester SR11481 was not as effective (IC_50_ 45.03 μM), possibly due to the presence of the ester bond (Fig 4B). A renilla luciferase reporter was cotransfected for normalization. To determine whether the inhibitory effect of the mimetics is limited to TNF-α-mediated induction of IKK/NF-κB, LPS-mediated NF-κB activation was examined. NF-κB activity was induced in Raw 264.7 by 1 μg/ml of LPS for 2 h, and the IKKi VII (2 μM) and the 8K-NBD peptide (400 μM) were included as positive controls. Expression of the NF-κB target genes cyclooxygenase 2 (COX-2), IL-6, IL-1β, TNF-α, IκBα, and inducible nitric oxide synthase (iNOS) were determined by quantitative real-time polymerase chain reaction (qRT-PCR) analysis. SR12460 and SR12454, which are more similar in structure than the other 2 mimetics, were able to significantly inhibit the transcription of all NF-κB target genes tested (Fig 4C). SR12343 displayed a similar profile to the 8K-NBD peptide, showing significant inhibition on COX-2, IL-6, and iNOS expression at a much lower concentration (50 μM) compared to NBD peptide (400 μM). SR11481 did not induce a detectable suppression of the NF-κB target genes, likely due to its poor stability. IKKi VII, while able to inhibit most NF-κB target gene expression, failed to down-regulate iNOS expression, which was significantly inhibited by the NBD peptide and all non-ester NBD mimetics. This suggests that IKK inhibitors targeting the ATP-binding pocket likely down-regulate the expression of a slightly different set of NF-κB regulated genes.

**Table 1:** The IC_50_ of NBD mimetics. Inhibitory effects of NBD mimetics on NF-κB activation were measured by luciferase assays at multiple concentrations: 0, 25, 50, 100, and 150 μM. IC_50_ of NBD mimetics was determined based on the dose-dependent curve by using GraphPad.

NBD Mimetics	IC_50_ (μM)
SR12460	11.34
SR12454	20.24
SR12343	37.02
SR11481	45.03

Abbreviations: IC_50_, half maximal inhibitory concentration; NBD, NEMO-binding domain; NEMO, NF-κB essential modulator; NF-κB, nuclear factor κB.

To confirm the qRT-PCR results, the levels of IL-6 production in Raw 264.7 cells activated by LPS were analyzed by ELISA. SR12460 and SR12454 were able to inhibit IL-6 secretion significantly in a dose-dependent manner (Fig 4D). Similarly, SR12343 inhibited IL-6 production, but was not as effective as SR12460 and SR12454, consistent with its higher IC_50_ in HEK293 cells. Although SR11481 failed to inhibit IL-6 significantly at the mRNA level, there was a significant reduction in the accumulation of IL-6 protein at 24 h. Moreover, SR11481 appears to be more effective at a lower concentration of 25 μM versus 50 μM.

### NBD mimetics target the NEMO/IKKß interaction to inhibit NF-κB signaling

**Fig 5:**
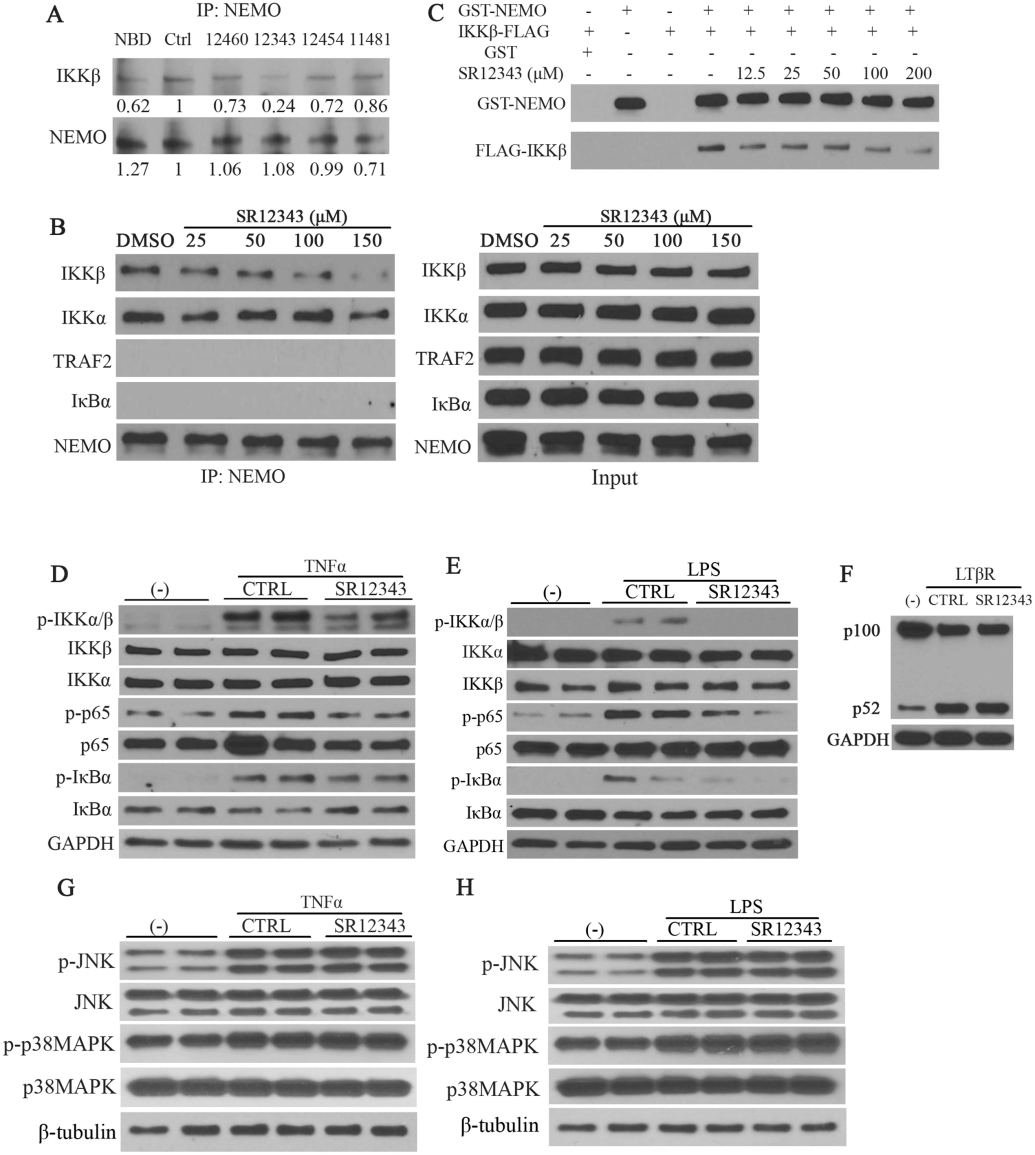
NBD mimetics selectively inhibit canonical NF-κB signaling by targeting the association of NEMO with IKKβ. (A) Co-IP analysis detecting the IKKβ/NEMO interaction. Raw 264.7 cells were pretreated with the indicated drugs, DMSO, 8K-NBD peptide (400 μM), SR12460 (100 μM), SR12343 (100 μM), SR12454 (100 μM), and SR11481 (100 μM) for 30 min, and the cells were then harvested for Co-IP. NEMO was probed as a loading control. (B) Raw 264.7 cells pretreated with SR12343 at indicated concentrations (0, 25, 50, 100, and 150 μM) for 30 min were subjected to Co-IP assay. NEMO-binding products were then analyzed for levels of IKKβ, IKKα, TRAF2, and IκBα (negative control), and levels of NEMO were used as a loading control (left panel). Right panel shows levels of proteins in input controls (10% of cell extract used for Co-IP). (C) GST-NEMO (15 nM), preincubated with inhibitors at indicated concentrations, was incubated with IKKβ-FLAG (15 nM) for 30 min at 30 °C and isolated using Glutathione argarose. The levels of IKKβ-FLAG binding to GST-NEMO were determined by western blot analysis with GST-NEMO used as a loading control. (D, E) Raw 264.7 cells pretreated with vehicle control or SR12343 (150 μM) for 30 min were stimulated with or without 10 ng/ml TNF-α (D) or 1 μg/ml LPS (E) for 10 min. Cell lysates were analyzed for activity of the IKK/NF-κB signaling pathway including activation of IKK complex, IκBα, and p65. GAPDH was used as a loading control. (F) Raw 264.7 cells were incubated with vehicle control or SR12343 (100 μM) for 30 min, followed by stimulation with or without anti-LTβR for 8 h. Cell lysates were harvested and analyzed for activation of noncanonical NF-κB signaling, the processing of p100 to p52. (G, H) Analysis of the effects of SR12343 (150 μM) on phosphorylation of JNK and p38MAPK in response to TNF-α (G) or LPS (H), using the same cell lysate being used in panels D and E. Co-IP, co-immunoprecipitation; GST, glutathione S-transferase; IKK, IκB kinase; JNK, c-Jun N-terminal kinase; LPS, lipopolysaccharide; LTβR, lymphotoxin β receptor; MAPK, mitogen-activated protein kinase; NBD, NEMO-binding domain; NEMO, NF-κB essential modulator; NF-κB, nuclear factor κB; TNF, tumor necrosis factor; TRAF2, TNF receptor-associated factor 2.

To determine whether the novel NBD mimetics target the NEMO-IKKβ interaction in vivo, co-immunoprecipitations were performed using extracts from Raw 264.7 macrophages (Fig 5A). All 4 of the mimetics reduce the IKKβ-NEMO interaction as well as or better than the NBD peptide, with SR12343 being the most effective. SR12343 also reduced the association between NEMO and IKKβ in Raw 264.7 cells in a dose-dependent manner (Fig 5B). No interaction between NEMO and TNF receptor-associated factor 2 (TRAF2) or IκBα was observed under these conditions. Previous studies suggested that the NBD peptide also inhibits the interaction of NEMO with IKKα [27]. However, SR12343 had only a marginal effect on NEMO/IKKα binding only at the highest dose, suggesting that these inhibitors affect the NEMO/IKKα interaction with a much lower efficiency (Fig 5B). To demonstrate that SR12343—which was the most effective mimetic in disrupting the NEMO/IKKβ interaction in vivo—affects the NEMO/IKKβ interaction directly, in vitro glutathione S-transferase (GST) pull-down assays were performed using recombinant GST-NEMO and FLAG-IKKβ. As shown in Fig 5C, SR12343 was able to disrupt the interaction between GST-NEMO and FLAG-IKKβ even at a dose of 12.5 μM. To demonstrate that the reduction of NF-κB-mediated transcription upon stimulation of TNF-α and LPS (Fig 4) is not due to off-target effects, the activation of NF-κB signaling and mitogen-activated protein kinase (MAPK) pathways was examined by western blot analysis. The levels of phosphorylated IKK complex, IκBα, and p65 in response to TNF-α and LPS were all reduced by SR12343 (Fig 5D & 5E). Consistently, the degradation of IκBα was partially reduced (Fig 5D & 5E). However, there were no changes in the levels of phospho-c-Jun N-terminal kinase (p-JNK), p-p38MAPK, total JNK, and p38MAPK by treatment with SR12343 (150 μM) in response to TNF-α or LPS stimulation (Fig 5G & 5H). These results suggest that the observed inhibitory effects of SR12343 are mediated directly through IKK/NF-κB but not due to off-target effects such as through the JNK or p38MAPK pathways. SR12343 treatment also had no effect on the activation of noncanonical NF-κB pathway by anti-lymphotoxin β receptor (LTβR) as shown by unaltered processing from p100 to p52 (Fig 5F).

### Novel NBD mimetics suppress LPS-induced acute pulmonary inflammation in vivo

**Fig 6:**
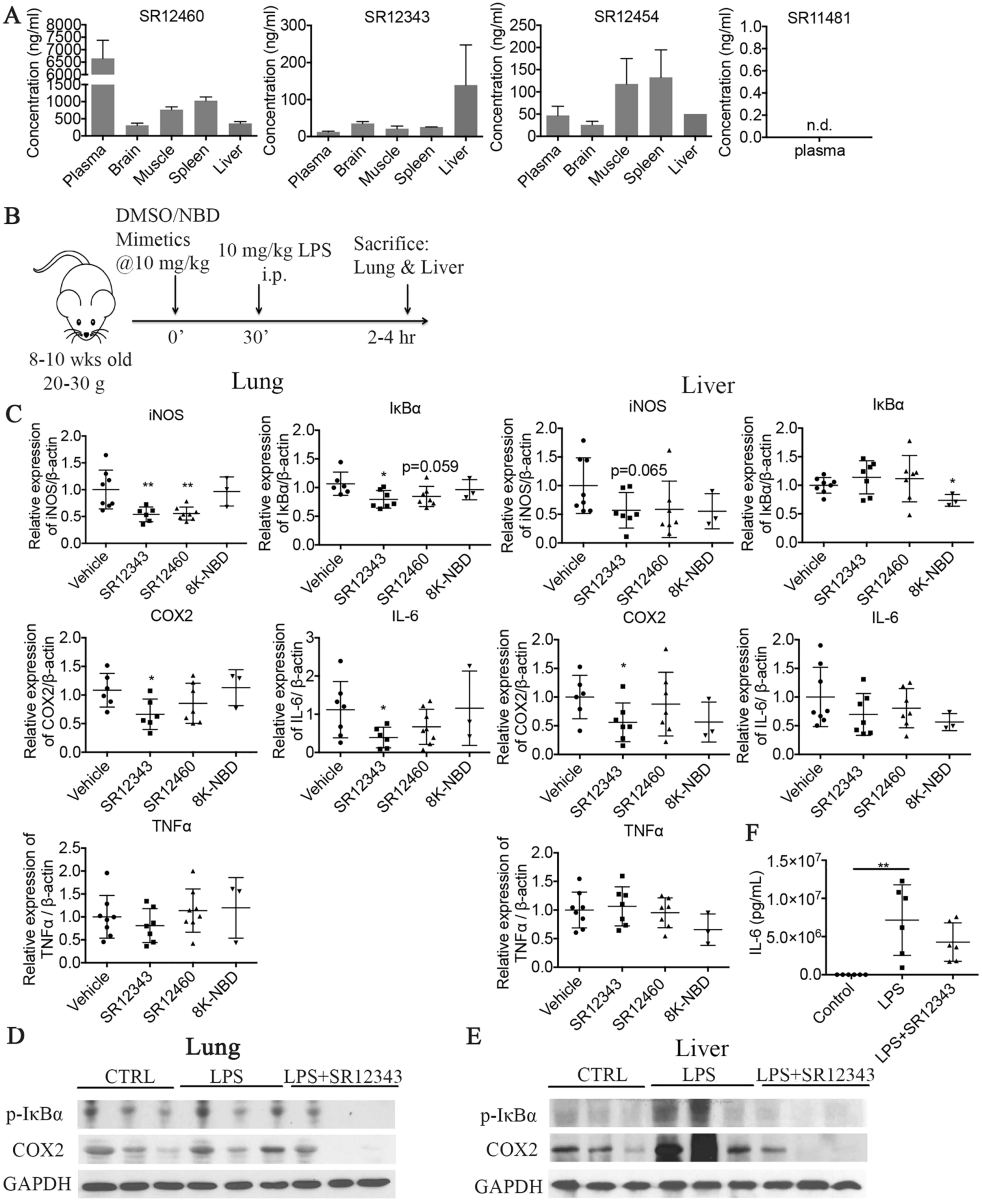
Newly identified NBD mimetics suppress LPS-induced acute inflammation in vivo. (A) The bioavailability property of SR12460, SR12343, SR12454, and SR11481. Concentrations of NBD mimetics were determined in plasma, brain, muscle, spleen, and liver 2 h post a single i.p. injection of 10 mg/kg of drugs; *n* = 3 per group. (B) Shown is the model to induce LPS-mediated acute inflammation and the treatment regimen with NBD mimetics. Mouse image via http://www.clker.com/clipart-mice-blank.html (copyright-free). (C) NBD mimetics suppressed LPS-induced acute inflammation in liver and lung by down-regulating NF-κB target gene expression. Vehicle, SR12343, SR12460, and 8K-NBD peptide were dosed at 10 mg/kg 30 min prior to the LPS treatment. Acute inflammation was induced by i.p. injection of 10 mg/kg LPS. Lung and liver were collected 2–4 h post injection, and mRNA was extracted for qRT-PCR analysis. (D, E) Western blot analysis was performed to probe for the expression of p-IκBα and COX-2 at protein level in liver and lung tissues. (F) Serum levels of IL-6 were determine by ELISA. *n* = 3–8 each group. *P < 0.05, **P < 0.01. Underlying data can be found in S1 Data. COX-2, cyclooxygenase 2; IL-6, interleukin 6; i.p., intraperitoneal; LPS, lipopolysaccharide; NBD, NEMO-binding domain; n.d., nondetectable; NEMO, NF-κB essential modulator; NF-κB, nuclear factor κB; qRT-PCR, quantitative real-time polymerase chain reaction.

To determine the stability of the NBD mimetics in vivo, their levels were measured in the plasma of mice 2 h post i.p. injection with 10 mg/kg of each compound. The concentration of SR12460 was very high in plasma (>6.5 μg/mL; 20 μM), while levels in brain, muscle, spleen, and liver were lower. SR12343 and SR12454 had lower plasma concentrations, with SR12343 showing a higher level in liver and SR12454 showing higher concentrations in muscle and spleen. The level of SR11481 was undetectable in plasma and tissues (Fig 6A). Because SR12454 and SR12460 share similar chemical structures and SR11481 demonstrates poor in vivo exposure, SR12460 and SR12343 were selected for further in vivo analysis. However, it is important to note that SR12343 was not as orally active as SR123460 (S1B Fig).

To determine their NF-κB inhibitory effects in vivo, SR12343 and SR12460 were tested in an acute model of LPS-induced systemic endotoxemia. C57BL/6J mice were pretreated with vehicle control, 8K-NBD peptide, or NBD mimetics at 10 mg/kg for 30 min, followed by LPS induction at 10 mg/kg (Fig 6B). Lung and liver were harvested 2–4 h post treatment for qRT-PCR analysis of NF-κB target genes. SR12343 was able to significantly inhibit NF-κB transcriptional activity in lung, demonstrated by the inhibition of iNOS, IκBα, COX-2, and IL-6, while expression of TNF-α was unchanged (Fig 6C). The NF-κB inhibitory effects of the compounds were less effective in liver compared to lung, demonstrated by significant inhibition of only COX-2 expression (Fig 6C). Despite lower plasma and tissue concentrations of SR12343 compared to SR12460, its inhibition of NF-κB/IKK in liver and lung were greater than that of SR12460 (Fig 6C). Similarly, acute treatment with SR12343 also affected the LPS-induced expression of NF-κB target genes at protein level. The extent of phosphorylation of IκBα and the levels of COX-2 were reduced in lung and liver tissues treated with SR12343 (Fig 6D and 6E). Serum levels of IL-6 detected by ELISA increased significantly following LPS induction and were reduced by acute treatment with SR12343 (Fig 6F). These results are consistent with our RT-PCR analysis shown in Fig 6C. However, no significant differences in lung histopathology were observed. Finally, there was a slight reduction in the number of white blood cells and neutrophils in the SR12343-treated group (Table 2). Taken together, the results demonstrate that SR12343 and SR12460 are effective at attenuating LPS-induced acute lung inflammation by suppressing NF-κB target gene expression.

**Table 2:** Hematological measures in acutely treated mice.

Parameters	Units	Vehicle (mean ± SD)	LPS (mean ± SD)	SR12343 (mean ± SD)
**Leukocytes**				
White blood cells	K/μL	6.9 ± 5.3	7.9 ± 4.6	7.4 ± 3.3
Neutrophils	K/μL	1.8 ± 2.1	2.3 ± 1.5	2.1 ± 1.3
Lymphocytes	K/μL	3.8 ± 2.2	4.4 ± 2.4	4.0 ± 1.3
Monocytes	K/μL	0.8 ± 0.4	0.5 ± 0.3	0.5 ± 0.2
Eosinophils	K/μL	0.3 ± 0.5	0.5 ± 0.3	0.6 ± 0.4
Basophils	K/μL	0.1 ± 0.1	0.2 ± 0.1	0.2 ± 0.2
Neutrophils	%	21.5 ± 9.9	27.7 ± 4.6	26.3 ± 8.0
Lymphocytes	%	60.8 ± 11.7	56.6 ± 5.6	57.7 ± 14.2
Monocytes	%	12.9 ± 3.5	6.3 ± 0.9	6.0 ± 0.7
Eosinophils	%	3.4 ± 2.8	6.9 ± 1.7	7.3 ± 3.7
Basophils	%	1.4 ± 0.8	2.5 ± 1.5	2.7 ± 2.0
**Erythrocytes**				
Red blood cells	M/μL	9.9 ± 1.1	10.6 ± 0.6	10.4 ± 0.3
Hemoglobin	g/dL	14.7 ± 0.8	15.2 ± 0.4	15.3 ± 0.4
Hematocrit	%	49.0 ± 2.2	52.3 ± 3.3	53.0 ± 1.5
Mean corpuscular volume	fL	49.7 ± 3.3	49.5 ± 0.9	51.2 ± 1.0
Mean corpuscular hemoglobin	pg	14.9 ± 0.8	14.4 ± 0.5	14.8 ± 0.2
Mean corpuscular hemoglobin concentration	g/dL	30.1 ± 0.4	29.2 ± 1.2	28.9 ± 0.2
Red cell distribution width	%	16.1 ± 1.1	16.0 ± 0.9	15.0 ± 0.2
**Thrombocytes**				
Platelets	K/μL	126.3 ± 58.8	133.7 ± 127.0	170.7 ± 64.0
Mean platelet volume	fL	5.8 ± 0.7	5.2 ± 0.4	5.7 ± 0.8

Abbreviations: LPS, lipopolysaccharide; SD, standard deviation.

### Novel NBD mimetics alleviate necrosis and muscle degeneration in mdx mice

**Fig 7:**
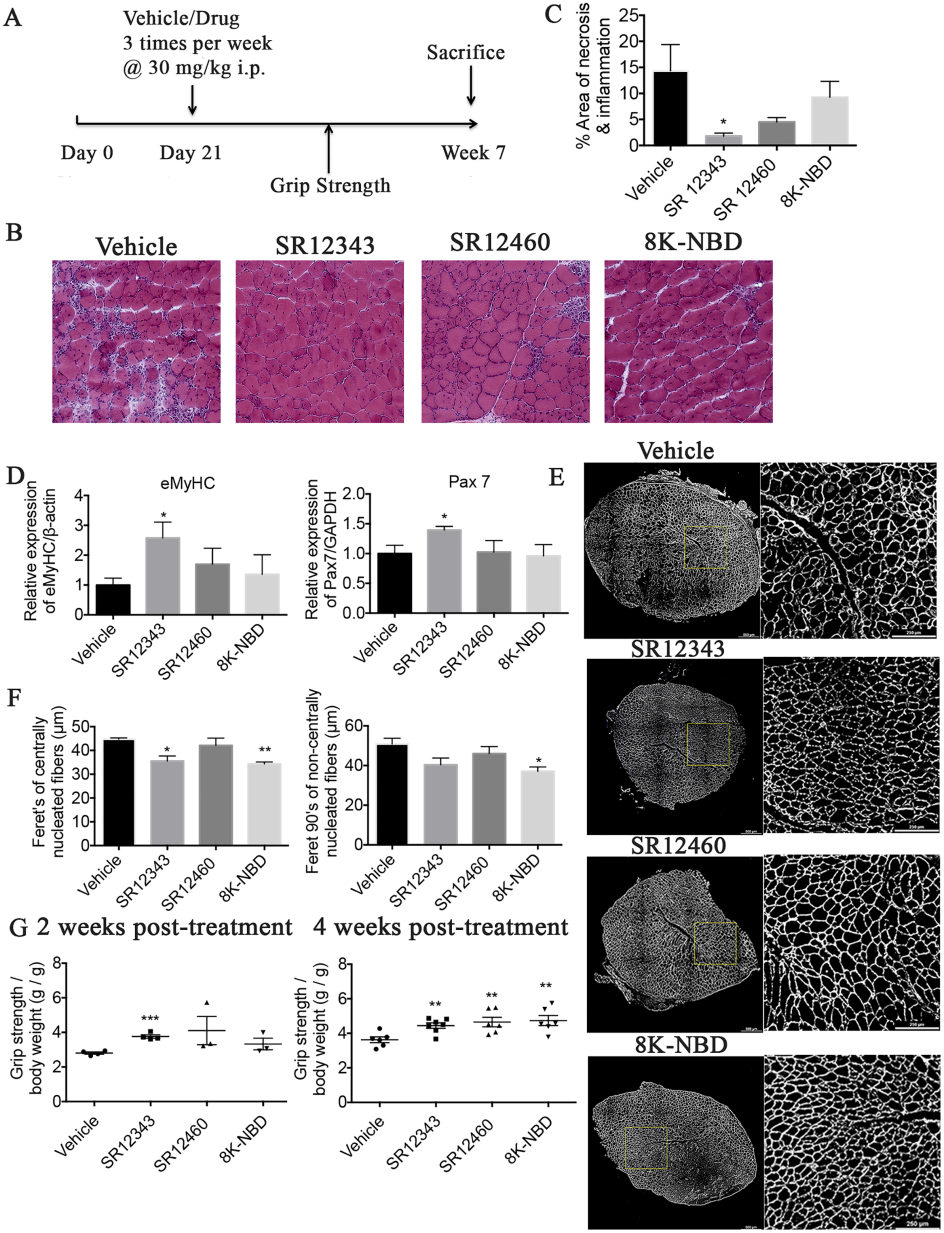
NBD mimetics improve muscular pathology and grip strength in mdx mice. (A) Shown is the treatment regimen with NBD mimetics in mdx mice. (B) Hematoxylin–eosin staining of TA muscle from 7-wk-old, treated or untreated mdx mice. Images were taken at magnification of 20×. Representative images were shown for each treatment group. (C) Quantitation of the percentage of area exhibiting necrosis or infiltration by using Image J. (D) qRT-PCR analysis of eMyHC and Pax7 in TA muscle. (E) TA muscle from 7-wk-old, treated or untreated mdx mice was stained with laminin by IHC to outline myofibers. Overall images of TA muscle were shown on the left side, and the inset panel images were shown on the right side. (F) Quantitation of minimum Feret diameter in centrally and noncentrally nucleated myofibers. (G) Forelimb force determined by grip strength test. Five-wk-old (2 wk post treatment) and 7-wk-old (4 wk post treatment) mdx mice, treated or untreated, were tested for forelimb grip strength. Five sequential tests were performed, and the force was normalized to body weight. Data were shown as mean +/− SEM. Dosages used are as follows: SR12343 (30 mg/kg), SR12460 (30 mg/kg), and 8K-NBD peptide (10 mg/kg). *n* = 6–7 each group. *P < 0.05, **P < 0.01. Underlying data can be found in S1 Data. eMyHC, embryonic myosin heavy chain; IHC, immunohistochemistry; NBD, NEMO-binding domain; NEMO, NF-κB essential modulator; Pax7, paired box protein 7; qRT-PCR, quantitative real-time polymerase chain reaction; TA, tibialis anterior.

Because SR12343 and SR12460 both reduced LPS-induced NF-κB activation in vivo, they were further tested in *mdx* mice, a mouse model of DMD in which NF-κB is chronically activated [28]. *Mdx* mice develop normally at birth and undergo a massive myonecrosis starting at 3 wk. Treatment of *mdx* mice with IKK/NF-κB inhibitors effectively reduces inflammation, blocks necrosis, and increases muscle regeneration. Initially, the effect of acute treatment of SR12343 on NF-κB DNA binding activity in tibialis anterior (TA) muscle in 9-wk-old *mdx* mice was examined by EMSA. Treatment with a single injection of 30 mg/kg of SR12343 resulted in a reduction in NF-κB DNA binding 2 h post injection (S4C Fig). Subsequently, *Mdx* mice were chronically treated with vehicle, SR12343 (30 mg/kg), SR12460 (30 mg/kg), or 8K-NBD (10 mg/kg) starting from day 21, 3 times/wk for 4 wk, similar to the dosing regimen used with the NBD peptide (Fig 7A) [7]. No significant weight loss was observed in chronically treated *mdx* mice (S3A Fig). In addition, there was no increase in levels of aspartate aminotransferase (AST), alanine aminotransferase (ALT), or alkaline phosphatase (ALP) in chronically treated *mdx* mice (S3B Fig), suggesting that treatment with SR12343 and SR12460 had no overt liver toxicity.

**Fig 8:**
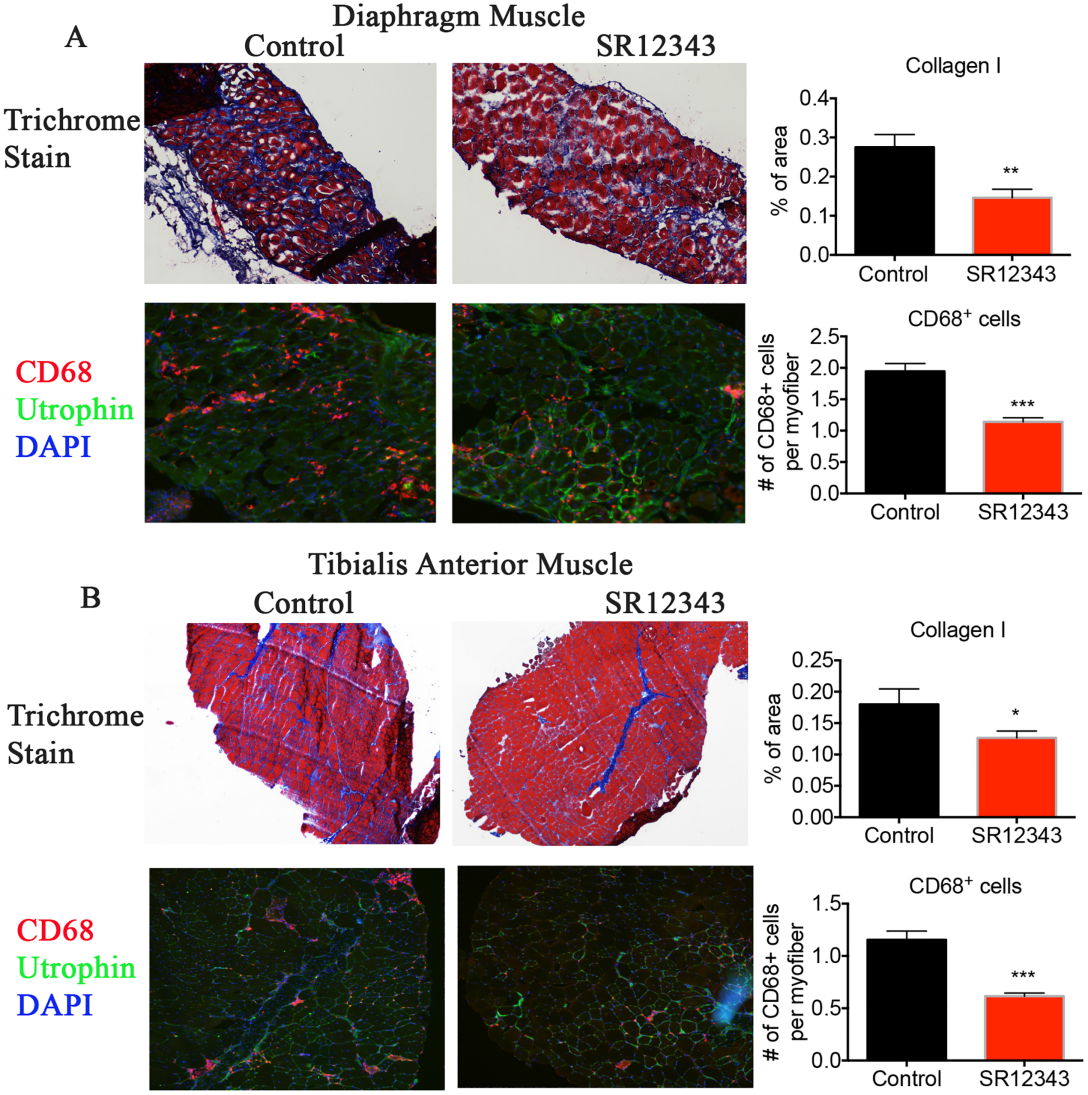
Chronic treatment with NBD mimetics reduces muscle fibrosis by decreasing macrophage infiltration in *mdx* mice. (A, B) Trichrome and CD68 staining of (A) diaphragm and (B) TA muscle from 7-wk-old, SR12343-treated or -untreated mdx mice. CD68 (red): marker of tissue macrophage; utrophin (green): stain for myofiber; DAPI (blue): nuclei. Trichrome images were taken at the magnification of 4× for TA and 10× for diaphragm. IHC images were taken at 10× for TA and 20× for diaphragm. Representative images were shown for each treatment group. Data were shown as mean +/− SEM. *P < 0.05; **P < 0.01; ***P < 0.001. Underlying data can be found in S1 Data. CD68, cluster of differentiation 68; IHC, immunohistochemistry; NBD, NEMO-binding domain; NEMO, NF-κB essential modulator; TA, tibialis anterior.

To determine whether SR12343 and SR12460 improve muscle pathology, TA muscle was stained with hematoxylin–eosin to assess inflammatory infiltration, necrosis, central nucleation, and fibrosis. Vehicle-treated TA muscles exhibited extensive infiltration and necrosis as reflected by clusters of inflammatory cells, disorganized myofibers, and nonuniform staining, with limited muscle regeneration. Consistent with previous studies, 8K-NBD peptide treatment reduced inflammatory cell infiltration and necrosis as shown in Fig 7B & 7C, while not significantly affecting the percentage of centralized myonuclei (S4A Fig) [29, 30]. SR12343 treatment led to the most significant pathological improvement, represented by limited infiltration and enhanced muscle reconstruction (Fig 7B & 7C and S4B Fig). Similarly, SR12460 treatment improved muscle pathology, although not as effectively as SR12343. In addition, Masson trichrome stain was conducted to further measure muscle fibrosis. Chronic treatment with SR12343 reduced muscle fibrosis (blue) in diaphragm and TA muscle tissues in comparison to control group (Fig 8A & 8B). To further quantitate the extent of inflammation in *mdx* muscle tissues, diaphragm and TA muscle were immunostained for cluster of differentiation 68 (CD68), a macrophage marker. Treatment with SR12343 reduced the number of CD68^+^ cells by almost 50% per myofiber in both diaphragm and TA tissues (Fig 8A & 8B). Notably, there is a higher level of macrophage infiltration in diaphragm in comparison to TA, as reflected by a greater number of CD68^+^ cells per myofiber. This result is consistent with the Trichrome stain in which the diaphragm exhibited more severe muscle fibrosis compared to TA. Moreover, qRT-PCR analysis revealed significant improvement of myofiber regeneration in SR12343-treated TA muscle relative to vehicle controls, evidenced by increased expression of embryonic myosin heavy chain (eMyHC) as well as paired box protein 7 (Pax7), a marker of skeletal muscle satellite cells (Fig 7D). SR12460 and the 8K-NBD peptide also increased eMyHC expression, but to a lesser extent than SR12343. To quantify the average fiber size in an unbiased way, TA muscle was stained with laminin to outline myofibers, and Feret diameter of all myocytes was quantitated in a whole muscle section. Fiber size of centrally and noncentrally nucleated myofibers was smaller in mice treated with SR12343 and the 8K-NBD peptide, suggesting an active reconstruction of myofibers during the regenerative phase (Fig 7E & 7F).

To determine whether the NBD mimetics improved muscle strength, grip strength was measured 2 wk and 4 wk post treatment to assess forelimb strength (Fig 7G). Compared to vehicle group, SR12343 significantly improved forelimb strength after 2 wk of treatment, indicating rapid muscle repair. All treatment groups, compared to control, displayed significantly strengthened forelimb strength 4 wk post treatment (Fig 7G). Collectively, the 2 lead compounds—SR12460 and, in particular, SR12343—markedly improved muscle function and muscular pathology in *mdx* mice.

## Discussion

The NBD peptide derived from the NBD in IKKβ is highly therapeutic in numerous mouse and canine models of inflammatory and degenerative diseases [6–9, 12, 13, 17, 18]. In fact, the NBD peptide is far more effective as a therapeutic than small-molecule inhibitors of the active site of IKKβ kinase. However, synthesis of the NBD peptide on a large scale can be problematic and extremely expensive, which impedes its clinical utility in humans, for whom a larger dose is needed compared to rodents and dogs. Also, the NBD peptide is not orally active. Thus, the development of small-molecule NBD mimetics represents a significant advance towards clinical translation of this established molecular target. Here, we identified and optimized several novel NBD mimetics that selectively inhibited IKK/NF-κB activation. The 2 lead compounds, SR12343 and SR12460, inhibited both TNF-α- and LPS-induced NF-κB activation more efficiently and at a lower concentration than the NBD peptide in both HEK293 and Raw 264.7 cells.

To identify the NBD mimetics, a computational screening campaign based on the pharmacophore model was used [21]. This structure-based pharmacophore model used has been utilized successfully to identify small-molecule inhibitors of p53 up-regulated modulator of apoptosis (PUMA) targeting its binding with B cell lymphoma 2 (Bcl-2) family members through the BH3 domain [31]. A crystal structure study of the NBD-IKKβ interaction demonstrated that amino acid residues W741, W739, and F734 are essential IKKβ hydrophobic motifs for dynamically interacting with NEMO [21]. Furthermore, a comprehensive analysis of binding energy hot spots revealed 3 regions critical for the protein–protein interaction interface between NEMO and IKKβ: the documented NBD regions (W739, W741, and L742) and 2 novel hot spot regions centered on IKKβ residues L708/V709 and L719/I723 [32]. Consistently, a mutated 11-mer NBD peptide (735–745)—with substitutions of arginine for W741 and W739—was unable to bind to NEMO [27]. A longer IKKβ-derived peptide (701–746) containing all 3 residues and domains exhibited the strongest affinity to NEMO, with IC_50_ around 10 nM, compared to the traditional 11-mer NBD peptide that was less potent, with IC_50_ around 100 μM [21, 32–34].

To identify NBD mimetics with greater bioactivity, F734 not contained within the 11–amino acid NBD peptide was included in our pharmacophore model and in silico screening. The novel NBD mimetics identified and optimized were able to inhibit NF-κB activity in a dose-dependent manner (IC_50_ approximately 10–40 μM) and were more potent than the 11-mer NBD peptide [21, 33]. These results are consistent with other results suggesting that, although IKKβ_737–742_ is the core component essential for IKK complex formation, the larger region spanning IKKβ_701–746_ enhances the binding affinity between IKKβ and NEMO [32, 34, 35].

The identified and optimized NBD mimetic SR12343 was able to dissociate the NEMO/IKKβ complexes both in vitro and in vivo (Fig 5), consistent with previous reports that NBD peptide blocks the association of preformed IKK complexes in inactive state [27, 33]. Also, although the NBD peptide has been reported to block interaction of NEMO with IKKα and reduce IL-1-induced, IKKα-dependent NF-κB activation in mouse embryonic fibroblasts (MEFs) [27, 36], SR12343 disrupted the binding of NEMO and IKKα much less effectively. This specificity for NEMO/IKKβ could be due to the fact that there is a methionine in IKKα at position 734 instead of a phenylalanine, while F734 along with W739 and W741 was included for the generation of the pharmocophore model. Our results also demonstrated that the SR12343 suppressed TNF-α- and LPS-induced NF-κB activity by inhibiting the phosphorylation of IKK, IκBα, and p65 while not affecting JNK and p38MAPK signaling (Fig 5). It appears that SR12343 has a more substantial inhibition in LPS-induced NF-κB activation in comparison to TNF-α stimulation. This is possibly due to a stronger secondary NF-κB activation upon TNF-α stimulation by up-regulating the expression of multiple pro-inflammatory factors. MAPK signaling can be stimulated by similar pro-inflammatory factors, such as TNF-α and LPS, increasing expression of pro-inflammatory genes via AP-1 and NFAT [1, 37]. In addition, SR12343 did not affect the noncanonical NF-κB pathway signaling through the LTβR (Fig 5F).

NBD mimetics inhibited a unique subset of target genes compared to IKK kinase inhibitors, particularly iNOS both in vitro and in vivo. The expression of iNOS in response to LPS can be down-regulated 6-fold in Raw 264.7 cells and by 50% in both lung and liver, by the treatment of mice with NBD mimetics. iNOS is involved in arginine metabolism, leading to the production of citrulline and nitric oxide (NO), the latter of which—acting as a free radical—promotes cytotoxicity and tissue injury [38]. iNOS-null *mdx* mice have significantly reduced macrophage cytolysis and decreased myofiber injury at both acute, necrotic phase (4 wk) and regenerative phase (6–12 wk), suggesting a critical role of NO-mediated myonecrosis in the pathology of *mdx* mice [38]. We demonstrated a reduction in necrosis, fibrosis, and inflammatory infiltration in both the diaphragm and TA muscle in treated *mdx* mice—in particular, SR12343-treated *mdx* mice—compared to controls. Also, similar to *mdx* mice treated with NBD peptide, treatment with the NBD mimetics—SR12343, in particular—resulted in increased myogenesis as shown by higher expression of eMyHC and Pax7 and the smaller size of centrally nucleated fibers (Fig 6). This is consistent with the observed ability of NF-κB to regulate cell differentiation via its transcriptional regulation of cyclin D1 [39]. Taken together, our results suggest that small-molecule NBD mimetics can simultaneously inhibit pro-inflammatory responses, reduce macrophage cytotoxicity, and improve muscle degeneration, with an even greater efficacy than NBD peptide. No overt signs of liver toxicity from the chronically treated mice were observed, based on serum clinical chemistries.

We previously demonstrated that chronic treatment of the *Ercc1*^−/Δ^ mouse model of accelerated aging with the NBD peptide delayed the onset of numerous age-related symptoms, improved pathology, and reduced cellular senescence. Similar to the NBD peptide, preliminary experiments suggest that chronic treatment of *Ercc1*^−/Δ^ mice with SR12343 resulted in an extended health span. Thus, these novel NBD mimetics could be used not only for treatment of inflammatory and degenerative diseases but also for aging.

Collectively, our data demonstrate that the novel small-molecule NBD mimetics are potent and highly selective IKK inhibitors that act by disrupting the association of IKK complexes. They exhibit significant inhibitory effects on NF-κB activation in the model of LPS-induced acute lung injury (ALI) and the murine model of DMD (*mdx* mice), suggesting the potential of NBD mimetics to become a distinct class of anti-inflammatory drugs. Taken together, NBD mimetics may provide therapeutic value for the chronic management of inflammatory diseases and cancers in the future.

## Methods

### Ethics statement

The animal studies were reviewed and approved by the Scripps Florida Institutional Animal Care and Use Committee (protocols #15–017 and 15–020) and were in compliance with the U. S. HHS Guide for the Care and Use of Laboratory Animals. The day-to-day care of animals was managed by the Scripps Florida Animal Research Center. Scripps Florida has an Animal Welfare Assurance on file with the Office of Laboratory Animal Welfare (OLAW), National Institutes of Health. The assurance number is #A4460-01, effective January 30, 2009. Scripps Florida’s registration under USDA regulations is certificate 93-R0015, effective December 5, 2005. The Association and Accreditation of Laboratory Animal Care International (AAALAC) awarded Scripps Florida full accreditation on June 25, 2008, and continuation was awarded on November 9, 2011. Mice were euthanized at 7 wk of age by carbon dioxide inhalation.

### Cells and mice

HEK293 cells and MEFs were grown in Dulbecco’s Modified Eagle Medium (with 4.5 g/L glucose and L-glutamine), supplemented with 10% fetal bovine serum, penicillin, and streptomycin. Raw 264.7 cells were cultured in RPMI-1640 media containing 10% heat-inactivated fetal bovine serum, penicillin, and streptomycin. C57BL/10ScSn-*Dmd*^*mdx*^/J and female C57BL/6 mice were purchased from the Jackson Laboratory. Mice were housed in the animal facilities of Scripps Florida under constant temperature and humidity. Animal protocols used in this study were approved by Scripps Florida Institutional Animal Care and Use Committee. Three-wk-old sex-matched *mdx* mice were dosed with SR12343 (30 mg/kg), SR12460 (30 mg/kg), 8K-NBD peptide (10 mg/kg), or vehicle by i.p. injection 3 times per wk for 4 wk. Mice were euthanized at 7 wk of age by carbon dioxide inhalation, and TA muscle was collected for histological analysis.

### 8K-NBD peptide and small molecules

8K-NBD (KKKKKKKKGGTALDWSWLQTE) peptide was synthesized at the peptide core facility of the University of Pittsburgh. For i.p. injections, the peptide was dissolved in 10% DMSO in PBS. The NBD mimetics were formulated in 10:10:80 of DMSO:Tween 80:Water for in vivo administration. ZINC small molecules were purchased from Enamine. All stock solutions for in vitro experiments were prepared in DMSO at 40 μM.

### LPS-induced acute lung inflammation

LPS (strain O111:B4) was prepared in PBS at a sublethal dose of 10 mg/kg. Female WT mice 8 to 10 wk old (20–30 g) were dosed by i.p. injection with vehicle, NBD peptide (10 mg/kg), or small molecules (10 mg/kg) for 30 min, followed by i.p. injection of saline (1 mL/kg) or LPS (10 mg/kg). Mice were euthanized 2 to 4 h post treatment, and lung and liver tissues were collected for further analysis.

### Functional grip strength analysis

Seven-wk-old treated or untreated *mdx* mice were measured for forelimb grip strength by using a digital grip strength meter paired with a metal grid (Bioseb, Vitrolles, France). Mice were allowed to grip the metal grid tightly, and readings were obtained by gently pulling the tail backward until release. Five sequential measurements were performed, and the average force was calculated.

### Firefly luciferase assay

HEK293 cells stably transfected with luciferase reporter plasmid driven by NF-κB were seeded in 96-well plates in triplicate and pretreated with DMSO or varying small molecules at indicated concentration for 30 min, followed by the stimulation of 10 ng/ml of TNF-α for 3 h [25]. Cells were washed with PBS once and harvested in Passive Lysis Buffer (Promega, Madison, WI). Luciferase assay (Promega) was performed by using a luminometer according to the manufacturer’s instructions.

### Dual-luciferase reporter assay

HEK293 cells grown in 10 cm plates were cotransfected with a coreporter of a Renilla plasmid driven by SV40 (Promega) and a firefly luciferase plasmid driven by NF-κB at the ratio of 1:3 with Lipofectamine 2000 (Invitrogen, Carlsbad, CA). Transiently transfected HEK293 cells were grown and treated as described above and subjected to a dual-luciferase reporter assay according to the manufacturer’s instruction. Briefly, NF-κB firefly luciferase and Renilla luciferase activity were detected sequentially. The relative luciferase activity was calculated by normalizing NF-κB luciferase to Renilla luciferase activity.

### MTT assay

HEK293 cells were grown in a 96-well plate at 3 × 10^4^ cells/well in triplicate and treated with DMSO or listed small molecules at indicated concentrations for 24 h. Cell survival was determined by adding 20 μl of 5 mg/ml MTT (thiazolyl blue tetrazolium bromide) to each well, followed by incubation in 37 °C for 3 h. Media were removed, and purple formazan was dissolved in 100 uL of DMSO. Absorbance was measured at 590 nm on a microplate reader (PerkinElmer, Waltham, MA). Cell viability was calculated by normalizing values to untreated controls.

### Western blotting

Cell lysate was prepared in RIPA buffer (20 mM Tris-HCl [pH 7.5], 150 mM NaCl, 1 mM Na^2^EDTA, 1 mM EGTA, 1% NP-40, 1% sodium deoxycholate, 2.5 mM sodium pyrophosphate, 1 mM beta-glycerophosphate, 1 mM Na_3_VO_4_, and 1 μg/ml leupeptin), supplemented with 1X protease inhibitor cocktails (MilliporeSigma, St. Louis, MO) and 1X Halt phosphatase inhibitor cocktail (ThermoFisher, Waltham, MA). Protein concentrations were determined by Lowry protein assays, and 30 μg of protein was resolved by MINI-PROTEAN TGX 4%–15% SDS-PAGE gels. Blots were incubated in primary antibodies overnight at 4 °C and secondary antibodies at room temperature for 1 h. Reagents and antibodies used are as follows: recombinant murine TNF-α (10 ng/mL; PeproTech #315-01A), LPS from *Escherichia coli*, serotype O111:B4 (1 μg/mL; Enzo #ALX-581-012-L001), recombinant murine IL-1α (0.5 ng/mL; PeproTech #211-11A), anti-LTβR antibody (1:300; Abcam #ab65089), anti-p-IκBα (1:1000; CST), IκBα (C-21) antibody (1:1000; #sc-371), p-IKKα/β (Ser176/180) antibody (1:500; CST #2697), IKKα antibody (1:1000; CST #2682), anti-IKKβ (1:1000; CST #8943), p-p65 (Ser536) (93H1) antibody (1:2000; CST #3033), p65 (D14E12) antibody (1:5000; CST #8242), p100/p52 antibody (1:500; CST #4882), p-TAK1 (Thr184/187) antibody (1:500; CST #4508), and anti-GAPDH (1:5000; CST #5174).

### Co-immunoprecipitation of endogenous IKKβ and NEMO

Raw 264.7 cells were seeded at 1 × 10^6^ cells/well in 6-well plates or 6 × 10^6^ cells/10 cm plate and pretreated with vehicle, small-molecule inhibitors, or NBD peptide for 30 min. Cells lysates were harvested in NP-40 lysis buffer supplemented with 1X protease inhibitor cocktails (Sigma). Proteins were immunoprecipitated by incubating 150 μg of lysates (1 μg/μl) with 10 μl of agarose-conjugated NEMO (FL-419) antibody (sc-8330 AC, Santa Cruz, Dallas, TX) on a rotary shaker at 4 °C for 4 h. Alternatively, we incubated 500 μg protein lysates with 10 to 15 μl of NEMO (FL-419) antibody (Santa Cruz) for 2 h at 4 °C, then added 50 μl Dynabeads (ThermoFisher) to the Ab-Ag complex and incubated on a rotator for 1 h at 4 °C. Ag-Ab-Bead complex was then washed with NP-40 buffer 3 times and PBS once. Protein was then denatured in SDS-sample buffer and resolved by MINI-PROTEAN TGX 4%–15% SDS-PAGE. Ten percent of cell lysates were probed as input controls for co-immunoprecipitation. Antibodies used are as follows: anti-IKKβ (1:1000; CST #8943), anti-NEMO (1:1000; CST #2685), TRAF2 (C192) antibody (1:1000; CST #4724), IKKα antibody (1:1000; CST #2682), and IκBα (C-21) antibody (1:1000; #sc-371).

### IKKβ-NEMO binding assay

Recombinant full-length NEMO tagged with GST at the N-terminus (SignalChem, Richmond, British Columbia, Canada) at 15 nM were preincubated with inhibitors for 15 min at RT, followed by incubation with 15 nM of recombinant full-length IKKβ tagged with FLAG at the C-terminus (Origene) for 30 min at 30 °C. TNT buffer (50 nM Tris [pH 7.5], 200 mM NaCl, 1% Triton X-100, and 1 mM DTT [pH 7.4]) was used as binding and washing buffer. Binding products were then incubated with 25 μl of glutathione agarose beads (ThermoFisher) for 1 h at 4 °C on a rotator. Final products were washed extensively with TNT buffer 4 times before the addition of sample buffer. Samples were then resolved by SDS-PAGE gels. Antibodies used are as follows: GST antibody (B-14) HRP (1:3000; #sc-138 HRP) and monoclonal anti-FLAG M2 antibody (1:2000; Sigma-Aldrich, F3165).

### EMSA

Cytoplasmic and nuclear fractions were extracted using the NE-PER nuclear and cytoplasmic extraction reagents (ThermoFisher) according to the manufacturer’s instructions. The gel shift assay was performed by following the previously described method [39]. In brief, 5 ug nuclear extract was incubated with 5X gel shift binding buffer for 10 min at room temperature (Promega). Next, an alpha-32P-deoxycytodine triphosphate-radiolabeled probe containing the consensus NF-κB binding sequence, at a concentration of 200,000 cpm/μL, was added to the reaction mix and incubated for 20 min at room temperature (MP Biomedicals, Santa Ana, CA). The reaction product was separated on a 6% nondenaturing polyacrylamide gel, prior to autoradiographic imaging. The oligonucleotide sequences are as follows: NF-κB template oligo, 5′-CAGGGCTGGGGATTCCCCATCTCCACAGTTTCACTTC-3′; NF-κB annealing oligo, 5′-GAAGTGAAACTGTGG-3′ (Integrated DNA Technologies, Coralville, IA).

### qRT-PCR

Snap-frozen tissues were preserved in RNAlater RNA stabilization solution (ThermoFisher). Total RNA was extracted from cells or tissues by using TRIZOL reagent (Life Technologies), and 1,500 ng of mRNA was subjected to synthesis of cDNA using SuperScript VILO cDNA synthesis kit. qRT-PCR was performed in a StepOnePlus Real-Time PCR system using Platinum SYBR Green qPCR SuperMix-UDG (ThermoFisher). Target gene expression was calculated using the comparative C_T_ method (ΔΔC_T_) by normalizing to an internal control gene Actb (β-actin). Primers used are as follows: *Ptgs2* (COX-2) forward: ACTCATAGGAGAGACTATCAAG; *Ptgs2* (COX-2) reverse: GAGTGTGTTGAATTCAGAGG; *Nfkbia* (IκBα) forward: CAGAATTCACAGAGGATGAG; *Nfkbia* (IκBα) reverse: CATTCTTTTTGCCACTTTCC; *Il1b* (IL-1β) forward: GGATGATGATGATAACCTGC; *Il1b* (IL-1β) reverse: CATGGAGAATATCACTTGTTGG; *Nos2* (iNOS) forward: TGAAATCCCTCCTGATCTTG; *Nos2* (iNOS) reverse: CCATGTACCAACCATTGAAG; *Tnf* (TNF) forward: CTATGTCTCAGCCTCTTCTC; *Tnf* (TNF) reverse: CATTTGGGAACTTCTCATCC; *Il6* (IL-6) forward: AAGAAATGATGGATGCTACC; *Il6* (IL-6) reverse: GAGTTTCTGTATCTCTCTGAAG. *Actb* (β-actin) forward: GATGTATGAAGGCTTTGGTC; *Actb* (β-actin) reverse: TGTGCACTTTTATTGGTCTC.

### Enzyme-linked immunosorbent assay

Raw 264.7 cells were grown in 96-well plates and pretreated with vehicle, IKKi VII (2 μM), and small molecules (at indicated concentration) for 1 h, followed by the stimulation with 1 μg/ml of LPS. Supernatant was collected 24 h later for ELISA analysis. IL-6 concentration was measured using a mouse IL-6 ELISA kit (BD) according to the manufacturer’s instructions.

The levels of TNF-α and IL-6 in mouse serum were detected using the Mouse TNF-α ELISA Kit (BD Biosciences, Franklin Lakes, NJ) and Mouse IL-6 ELISA Kit (BD Biosciences) per manufacturer’s specifications. The absorbance was quantified at 450 nm using a Spectramax i3 (Molecular Devices, San Jose, CA) plate reader. All standards and samples were measured in duplicate.

### Hematoxylin–eosin staining

Tissues fixed in 10% neutral buffered formalin (NBF) overnight were embedded in paraffin. Tissue was sectioned at 5 μm using a microtome. Hematoxylin–eosin staining was conducted following a standard protocol [30].

### Masson trichrome stain

Masson trichrome stain was performed with the Masson Modified IMEB Trichrome staining kit (IMEB, San Marcos, CA), as instructed by the manufacturer’s protocol, which stains collagen blue, muscle fibers red, and nuclei black. Briefly, the frozen tissue sections were fixed with 10% formalin and subsequently incubated in iodine solution, hematoxylin solution, Biebert’s Scarlet Acid Fuschin solution, Phospotungistic Phosphomolybdic Acid Solution, and Aniline Blue Stain Solution. Slides were then rinsed, dehydrated, and mounted for imaging.

### Muscle immunohistochemistry and quantification

To ensure rigorous quantitation of average fiber size, TA muscle was stained with laminin to outline myofibers. Using NIS Elements (Nikon, Melville, NY) analysis software, the laminin signal was corrected for background and underwent signal homogenization and structure amplification by utilizing a local contrast and kernel-based smoothing algorithm. Muscle fibers were defined by applying a uniform binary mask to the negative space of the laminin image, then segmented with the use of a Boolean operator to isolate only negative space overlapping with the total tissue area present in the image. Nuclei defined by Hoechst labeling (Sigma bisBenzimide H 33258) was defined using a uniform binary mask, and segmentation was performed with a watershed algorithm. Centrally nucleated fibers were determined by first eroding the binary mask of the muscle fibers so that only the central portion of each fiber was present. A Boolean operator was used to determine whether the eroded fibers overlapped with the nuclear-associated mask, and if so, they were considered to be centrally nucleated. Using the original muscle fiber mask, Feret diameters for centrally nucleated and noncentrally nucleated fibers were then measured.

The frozen tissue sections were fixed with 4% paraformaldehyde for 1 h and incubated with 1% BSA for blocking. The primary antibodies CD68 (Abcam, Cambridge, MA) and utrophin (Santa Cruz) were applied at 1:200 for 2 h. The secondary antibodies Alexa Fluor 594 and Alexa Fluor 488 were then applied at 1:400 for 1 h. DAPI (1;1000) solution was used to stain the cell nucleus. All the processes were performed at room temperature.

### Hematological and serum biochemical analysis

Nine mice (male; C57BL/6, 9–13 wk) were randomly divided into 3 groups with 3 mice in each group. Mice were injected i.p. with vehicle (DMSO:Tween80:H_2_O, 10:10:80) or small-molecule SR12343 (30 mg/kg) for 30 min and then injected i.p. with saline (1 ml/kg) or LPS (10 mg/kg in saline). After 2 h, mice were euthanized by CO_2_ asphyxiation prior to blood sampling via cardiac puncture. The blood from each mouse was put into 2 collection tubes with coagulant or anticoagulant heparin (approximately 0.6 mL each). The samples in the tubes coated with the anticoagulant heparin were hand-mixed several times and placed on wet ice for hematological analysis using an automatic hematology analyzer (Hemavet 950FS; Drew Scientific, Miami Lakes, FL). Samples in tubes containing no anticoagulant were allowed to clot before centrifugation and submission for serum biochemical analysis using the VetScan VS2 Analyzer with the Comprehensive Diagnostic Profile reagent rotor (Abaxis, Union City, CA). The ALT, AST, and ALT activities in the serum were quantitated by a colorimetric, enzymatic method using the Clinical Chemistry Analyzer Cobas c311 (Roche Diagnostics, Risch-Rotkreuz, Switzerland) as per the manufacturer’s instructions.

### Pharmacokinetics study

The pharmacokinetic profile of the NBD mimetics was determined in male C57BL/6J mice (*n* = 3). The drugs were formulated in 10:10:80 of DMSO:Tween 80:water and were dosed by i.p. injection at a final dose of 10 mg/kg. Blood, brain, muscle, spleen, and liver were collected 2 h post treatment and were analyzed by mass spectrometry by following a protocol previously described [40].

### Pharmacophore model generation

X-ray structure of the complex NEMO/IKKβ retrieved from the PDB (ID 3BRV) was used to generate a structure-based pharmacophore model [21]. The three-dimensional (3D) pharmacophore model was created with LigandScout [22, 23] and was based on interactions that define the protein–protein interaction, such as hydrophobic interactions, hydrogen bonding, and electrostatic interactions. Features identified by the LigandScout software are those that take into consideration chemical functionality but not strict structural topology or definite functional groups. As a result, completely new potential pharmacons can be identified through database screening. Moreover, to increase the selectivity, the LigandScout model includes spatial information regarding areas inaccessible to any potential ligand, thus reflecting possible steric restrictions. In particular, excluded volume spheres placed in positions that are sterically not allowed are automatically added to the generated pharmacophore model. In this way, the structure-derived pharmacophore model contains the pharmacophore elements of the candidate ligands in response to the protein’s active site environment [41].

### Similarity search

Recognition of small molecules by proteins is largely mediated by molecular surface complementarities. Structure-based drug design approaches use this as the fundamental guiding principle; that is, closely related molecules will elicit similar activity in a biological assay [42, 43]. The morphological similarity is a similarity technique dependent only on surface shape and charge characteristics of ligands [44]. Morphological similarity is defined as a Gaussian function of the differences in the molecular surface distances of 2 molecules at weighted observation points on a uniform grid. The computed surface distances include both distances to the nearest atomic surface and distances to donor and acceptor surfaces. This function is dependent on the relative alignment of the molecules, and consequently their alignment and conformation must be optimized. The conformational optimization problem is solved by fragmentation, conformational search, alignment, and scoring, followed by incremental reconstruction from high-scoring aligned fragments. The alignment problem is addressed by exploiting the fact that 2 unaligned molecules or molecular fragments that have some degree of similarity will have some corresponding set of observers that are seeing the same things. Optimization of the similarity of 2 unaligned molecules or molecular fragments is performed by finding similar sets of observers of each molecule that form triangles of the same size [41, 44].

### In silico ADME/Tox screening

Computational modeling tools were used to estimate the bioavailability, aqueous solubility, blood brain barrier potential, human intestinal absorption, the cytochrome P450 (i.e., CYP2D6) enzyme inhibition potential, mutagenicity, and hERG inhibition of the hits obtained from the database screening. The bioavailability, aqueous solubility, and human intestinal absorption were estimated using the ACD/ADME Boxes software (ACD Labs, Toronto, Canada; http://www.acdlabs.com), while mutagenicity, hERG, and CYP2D6 inhibition were estimated with ACD/Tox screening (ACD Labs, Toronto, Canada; http://www.acdlabs.com).

### Statistical analysis

All values were presented as mean +/− SEM or mean +/− SD. Microsoft Excel and Graphpad Prism 6 were used for statistical analysis. Two-tailed Student *t* test was performed to determine differences between 2 groups. When comparing differences in more than 2 groups, 1-way ANOVA (Dunnett test) was conducted. A value of *P* < 0.05 was considered statistically significant, shown as “*” for *P* <0.05, “**” for *P* <0.01, and “***” for *P* < 0.001.

## Supporting information

S1 Data Data for primary Figs 2, 3, 4, 6, 7, 8 and S1, S3 and S4 Figs. (XLSX)

S1 Fig IC_50_ and the complete pharmacokinetic profiles of NBD mimetics. (A) Dose-dependent curve of NBD mimetics ranging from 0 to 150 μM was determined by NF-κB luciferase assays in HEK293 cells. (B) Complete pharmacokinetic profiles of SR12460 and SR12343 administered i.v. or by oral gavage in mice. Underlying data can be found in S1 Data. AUC%Extrap, percentage of the area under the curve extrapolated to infinity from T_last_ to infinity; AUC_INF_obs_, area under the curve from 0 to infinity; AUC_last_, area under the curve from the time of dosing to the last measurable concentration; C_max_, maximum observed concentration; Cl_obs_, total serum clearance; HEK, human embryonic kidney 293 cells; IC_50_, half maximal inhibitory concentration; NBD, NEMO-binding domain; NEMO, NF-κB essential modulator; NF-κB, nuclear factor κB; T_1/2_, half-life; T_max_, time of maximum concentration.
(TIF)

S2 Fig SR12343 reduces NF-κB activation by disrupting phosphorylation of IKKα/β without affecting TAK1 phosphorylation. (A) SR12343 at indicated concentrations (0, 25, 50, 100, and 150 μM) inhibited phosphorylation of IKKα/β and p65 and prevented IκBα from degradation. (B) SR12343 reduced IL-1α-induced phosphorylation of TAK1 and IKKβ in IKKα^−/−^ MEFs. IKK, IκB kinase; IL-1, interleukin 1; MEF, mouse embryonic fibroblast; NF-κB, nuclear factor κB.
(TIF)

S3 Fig NBD mimetics display no effects on body weight in treated *mdx* mice. (A) Body weight was monitored in chronically treated *mdx* mice, and no significant differences were found. (B) Serum samples from chronically treated mdx mice were analyzed for levels of AST, ALT, and ALP, indicators of liver damage, using Clinical Chemistry Analyzer Cobas c311. Underlying data can be found in S1 Data. ALP, alkaline phosphatase; ALT, alanine aminotransferase; AST, aspartate aminotransferase; NBD, NEMO-binding domain; NEMO, NF-κB essential modulator.
(TIF)

S4 Fig Chronic treatment with NBD mimetics improves muscle pathology by inhibiting NF-κB DNA binding activity in *mdx* mice. (A) Quantification of percentage of centralized myonuclei. (B) Larger area of hematoxylin–eosin staining of TA muscles from different treatment groups. (C) EMSA analysis of NF-κB DNA binding activity in vivo was performed using extracts from TA tissues from *mdx* mice. Single dose of SR12343 at 30 mg/kg was given by i.p. TA muscles were harvested at 2 h post injection for EMSA analysis. Underlying data can be found in S1 Data. EMSA, electrophoretic mobility shift assay; i.p., intraperitoneal; NBD, NEMO-binding domain; NEMO, NF-κB essential modulator; NF-κB, nuclear factor κB; TA, tibialis anterior.
(TIF)

S1 Table Small-molecule derivatives selected from ZINC 10.0 database. (DOCX)

## Abbreviations

3D: three-dimensional
ADME/Tox: Absorption, Distribution, Metabolism, Excretion, and Toxicity
ALI: acute lung injury
ALP: alkaline phosphatase
ALT: alanine aminotransferase
AST: aspartate aminotransferase
Bcl-2: B cell lymphoma 2
CD68: cluster of differentiation 68
COX-2: cyclooxygenase 2
DMD: Duchenne muscular dystrophy
EMSA: electrophoretic mobility shift assay
eMyHC: embryonic myosin heavy chain
GRMD: golden retriever muscular dystrophy
GST: glutathione S-transferase
HEK293: human embryonic kidney 293 cells
i.p.: intraperitoneal
IC_50_: half maximal inhibitory concentration
IKK: IκB kinase
IL-1: interleukin 1
iNOS: inducible nitric oxide synthase
JNK: c-Jun N-terminal kinase
LPS: lipopolysaccharide
LTβR: lymphotoxin β receptor
MAPK: mitogen-activated protein kinase
MEF: mouse embryonic fibroblast
MTT: 3-(4,5-dimethylthiazol-2-yl)-2,5-dephenyltetrazolium bromide
n.d.: nondetectable
NBD: NEMO-binding domain
NEMO: NF-κB essential modulator
NF-κB: nuclear factor κB
NLS: nuclear localization sequence
NO: nitric oxide
Pax7: paired box protein 7
PDB: Protein Data Bank
p-JNK: phospho-c-Jun N-terminal kinase
PTD: protein transduction domain
PUMA: p53 up-regulated modulator of apoptosis
qRT-PCR: quantitative real-time polymerase chain reaction
RHD: Rel-homology domain
RMSD: root-mean-square deviation
SAR: structure–activity relationship
SD: standard deviation
TA: tibialis anterior
TCR: T-cell receptor
TNF-α: tumor necrosis factor α
TRAF2: TNF receptor-associated factor 2
