# Supplementary figures and images for: Development of novel NEMO-binding domain mimetics for inhibiting IKK/NF-κB activation
Source: PLoS Biol. 2018 Jun 11;16(6):e2004663. doi: 10.1371/journal.pbio.2004663 (PMC6013238; doi:10.1371/journal.pbio.2004663)

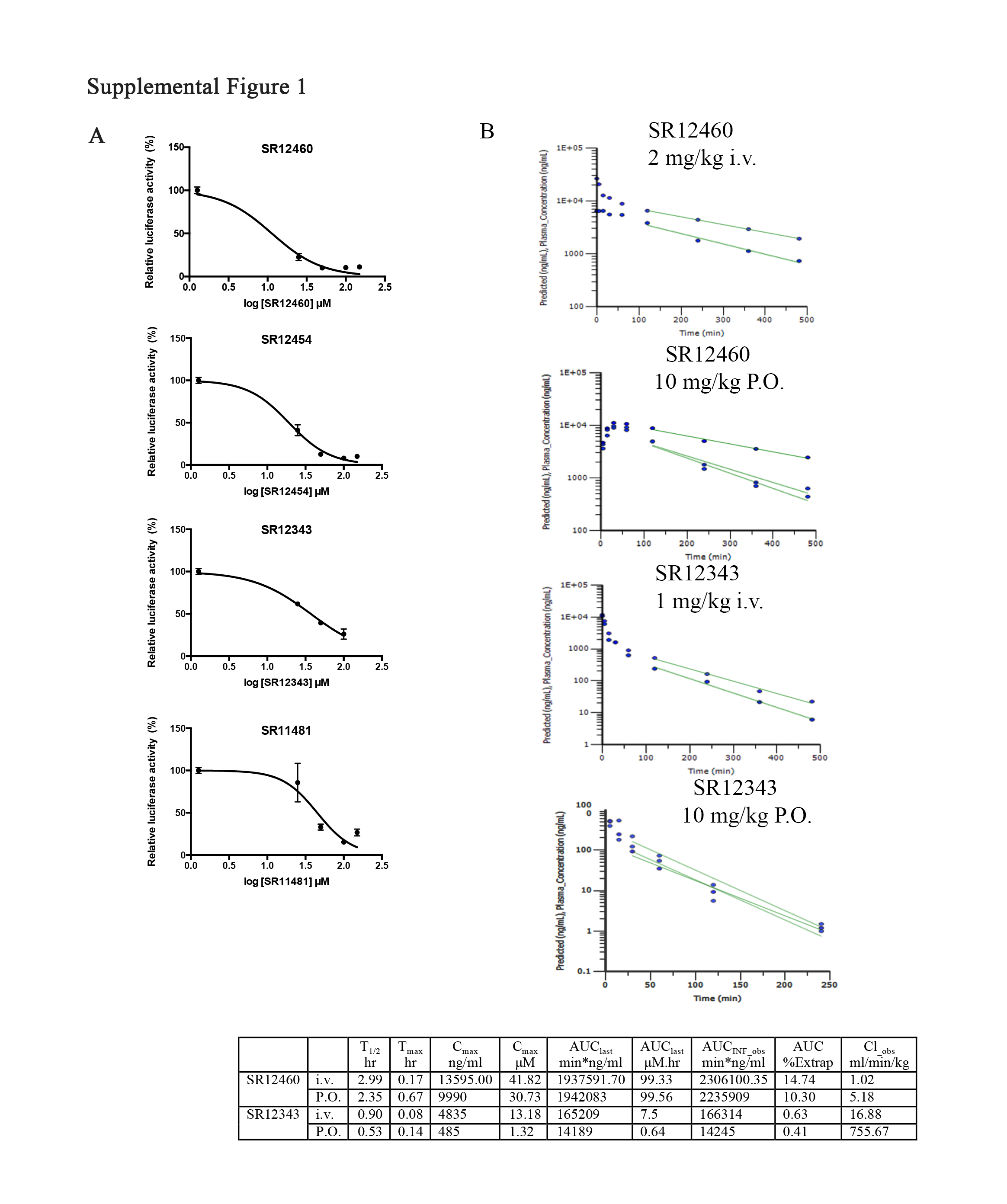

Supplement: S1 Fig — (A) Dose-dependent curve of NBD mimetics ranging from 0 to 150 μM was determined by NF-κB luciferase assays in HEK293 cells. (B) Complete pharmacokinetic profiles of SR12460 and SR12343 administered i.v. or by oral gavage in mice. Underlying data can be found in S1 Data. AUC%Extrap, percentage of the area under the curve extrapolated to infinity from Tlast to infinity; AUCINF_obs, area under the curve from 0 to infinity; AUClast, area under the curve from the time of dosing to the last measurable concentration; Cmax, maximum observed concentration; Clobs, total serum clearance; HEK, human embryonic kidney 293 cells; IC50, half maximal inhibitory concentration; NBD, NEMO-binding domain; NEMO, NF-κB essential modulator; NF-κB, nuclear factor κB; T1/2, half-life; Tmax, time of maximum concentration. (TIF) [file pbio.2004663.s002.tif]

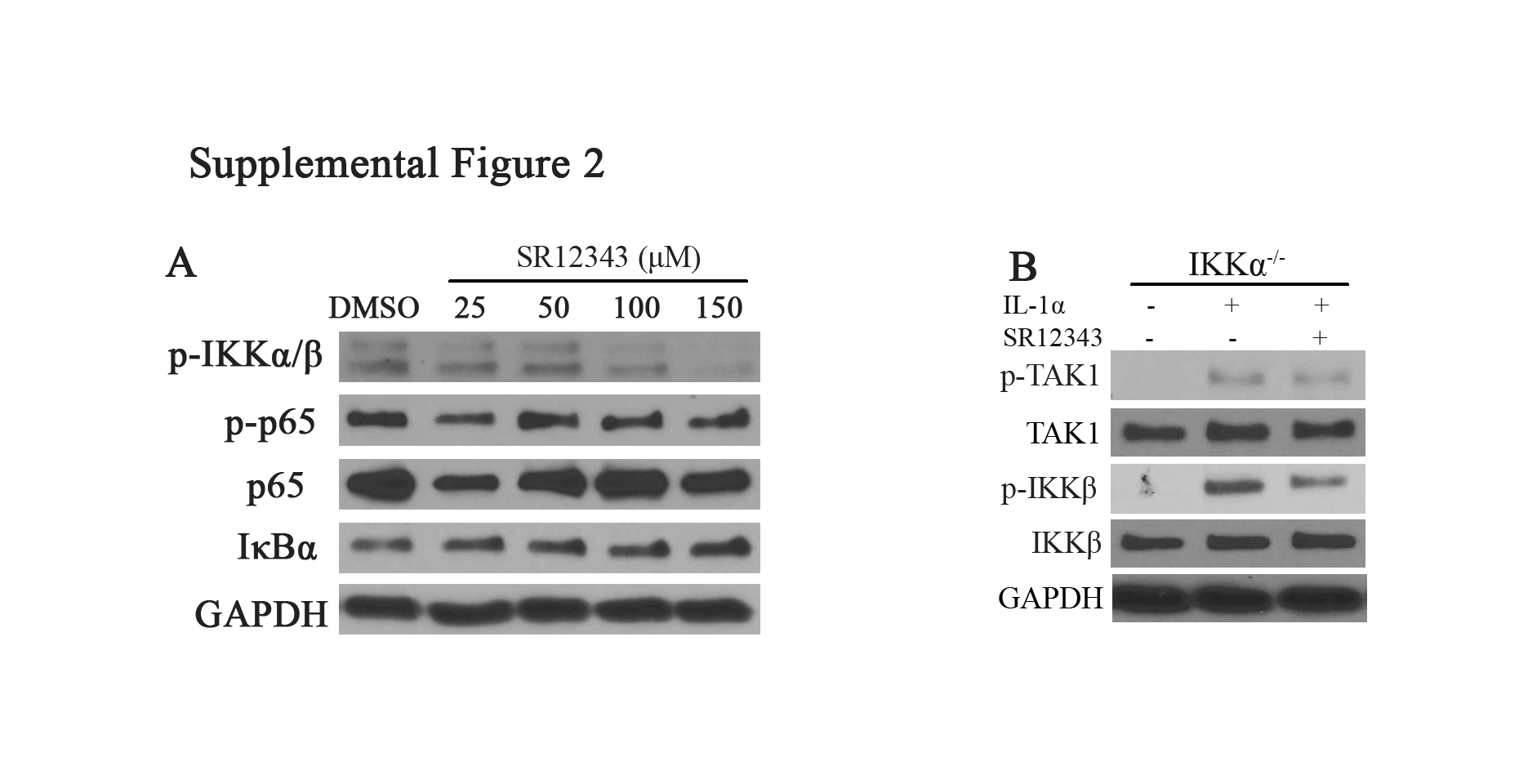

Supplement: S2 Fig — (A) SR12343 at indicated concentrations (0, 25, 50, 100, and 150 μM) inhibited phosphorylation of IKKα/β and p65 and prevented IκBα from degradation. (B) SR12343 reduced IL-1α-induced phosphorylation of TAK1 and IKKβ in IKKα−/− MEFs. IKK, IκB kinase; IL-1, interleukin 1; MEF, mouse embryonic fibroblast; NF-κB, nuclear factor κB. (TIF) [file pbio.2004663.s003.tif]

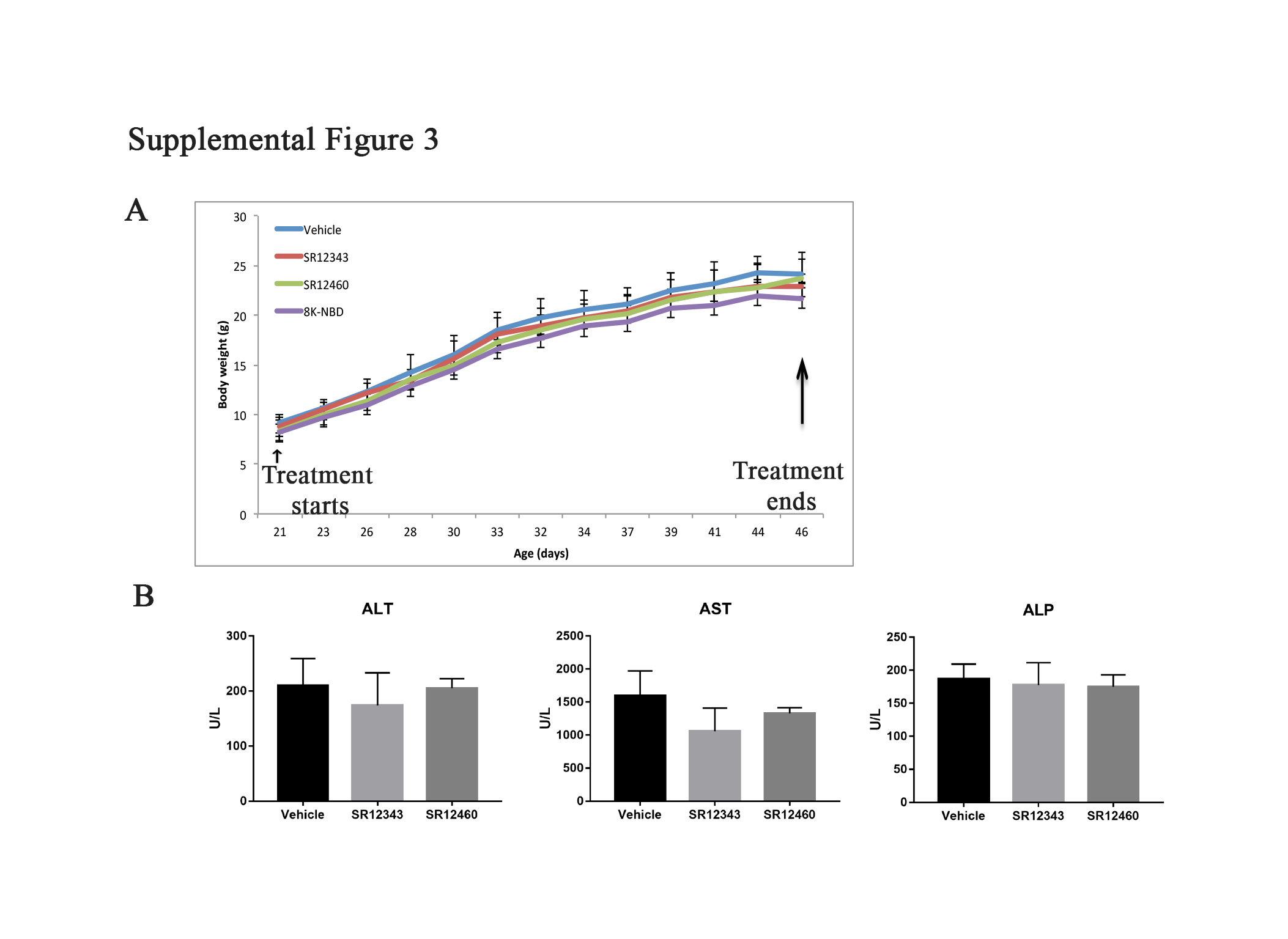

Supplement: S3 Fig — (A) Body weight was monitored in chronically treated mdx mice, and no significant differences were found. (B) Serum samples from chronically treated mdx mice were analyzed for levels of AST, ALT, and ALP, indicators of liver damage, using Clinical Chemistry Analyzer Cobas c311. Underlying data can be found in S1 Data. ALP, alkaline phosphatase; ALT, alanine aminotransferase; AST, aspartate aminotransferase; NBD, NEMO-binding domain; NEMO, NF-κB essential modulator. (TIF) [file pbio.2004663.s004.tif]

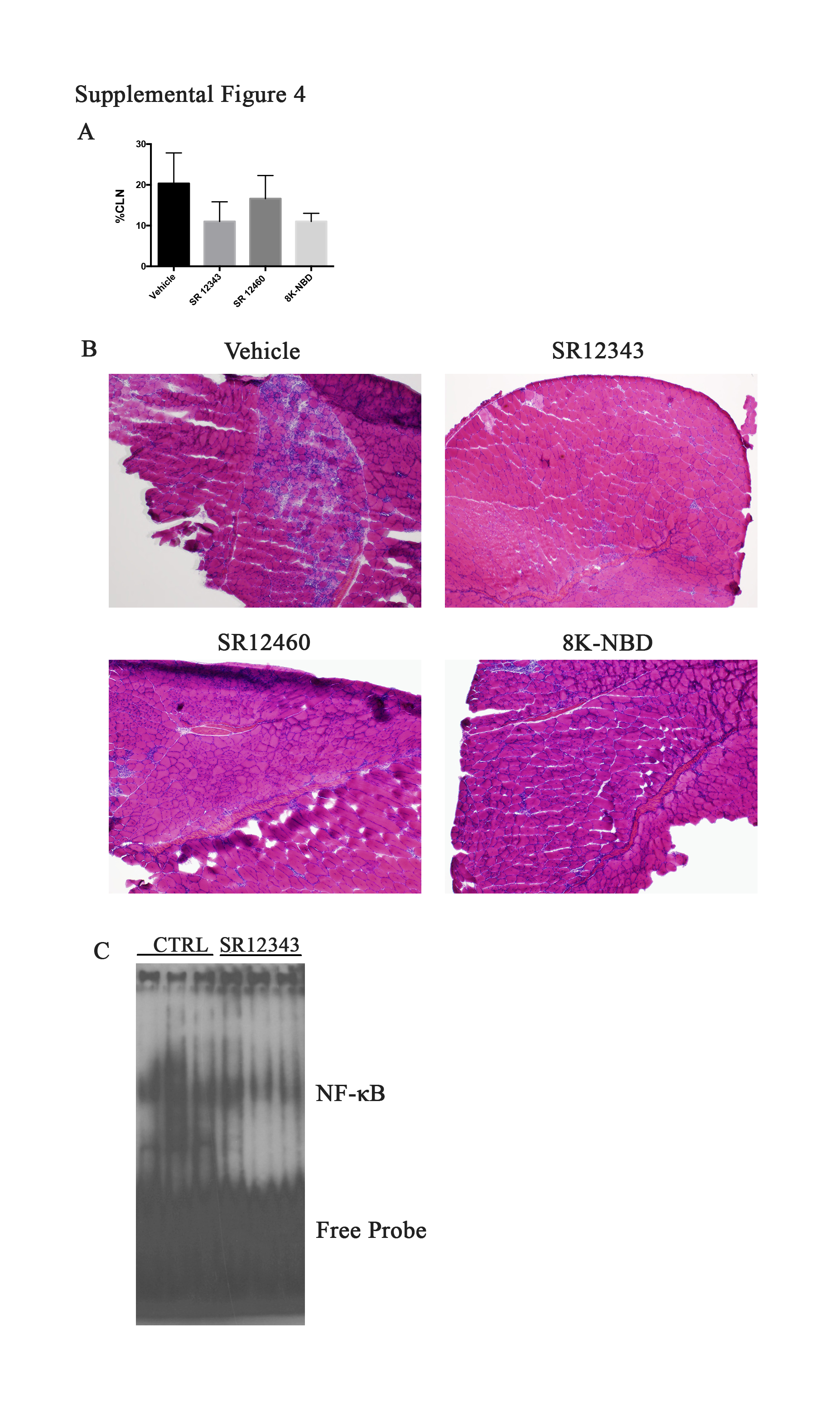

Supplement: S4 Fig — (A) Quantification of percentage of centralized myonuclei. (B) Larger area of hematoxylin–eosin staining of TA muscles from different treatment groups. (C) EMSA analysis of NF-κB DNA binding activity in vivo was performed using extracts from TA tissues from mdx mice. Single dose of SR12343 at 30 mg/kg was given by i.p. TA muscles were harvested at 2 h post injection for EMSA analysis. Underlying data can be found in S1 Data. EMSA, electrophoretic mobility shift assay; i.p., intraperitoneal; NBD, NEMO-binding domain; NEMO, NF-κB essential modulator; NF-κB, nuclear factor κB; TA, tibialis anterior. (TIF) [file pbio.2004663.s005.tif]
