# Supplementary material for: Development of novel NEMO-binding domain mimetics for inhibiting IKK/NF-κB activation
Source: PLoS Biol. 2018 Jun 11;16(6):e2004663. doi: 10.1371/journal.pbio.2004663 (PMC6013238; doi:10.1371/journal.pbio.2004663)
Supplement: S1 Table — (DOCX) [file pbio.2004663.s006.docx]

**S1 Table:** Small molecule derivatives selected from ZINC 10.0 data base.

| ZINC ID | Abbreviation | Popular name | Structure |
| --- | --- | --- | --- |
| 09642366 | ZINC1 | [(5-chloro-2-pyridyl)carbamoylmethyl](http://zinc.docking.org/synonym/%285-chloro-2-pyridyl%29carbamoylmethyl) | 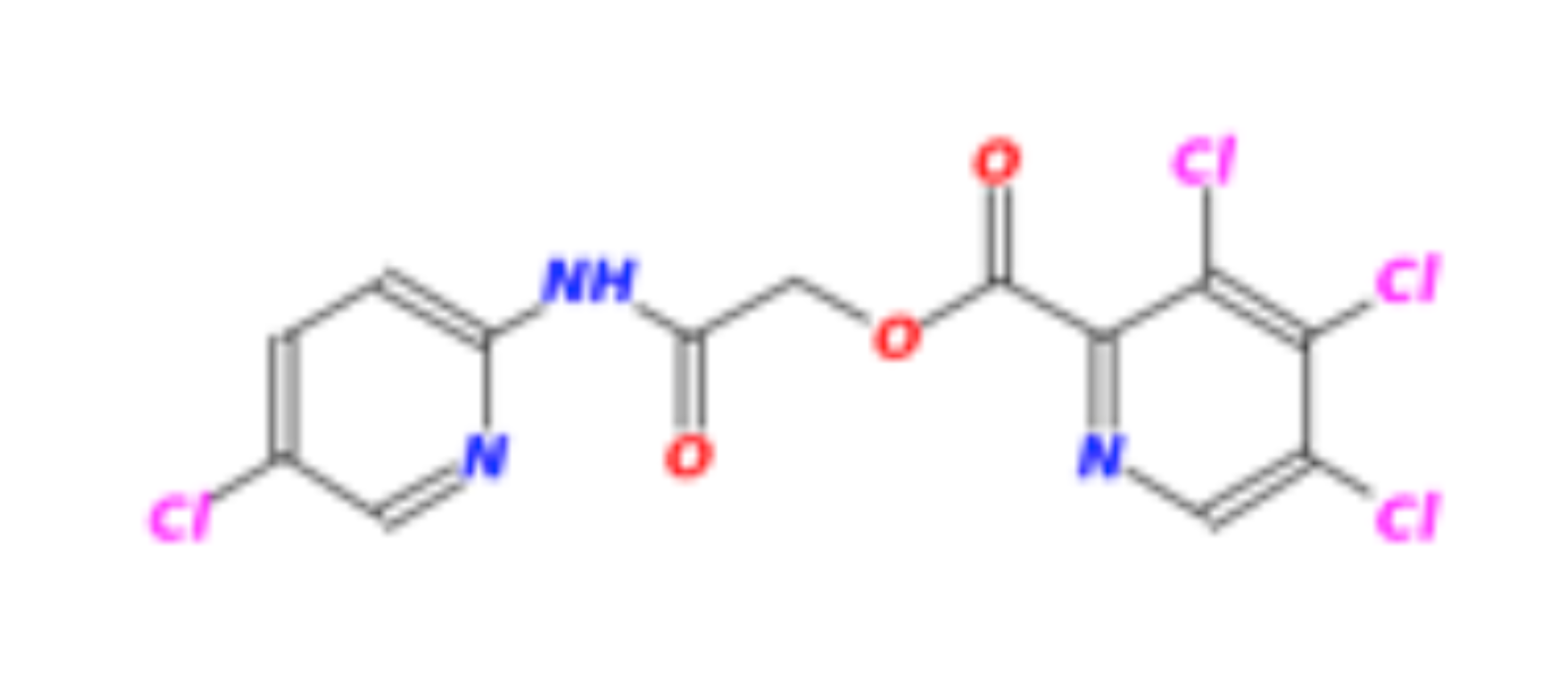 |
| 09645305 | ZINC2 | [(3,5-dichloro-2-pyridyl)carbamoylmethyl](http://zinc.docking.org/synonym/%283%2C5-dichloro-2-pyridyl%29carbamoylmethyl) | 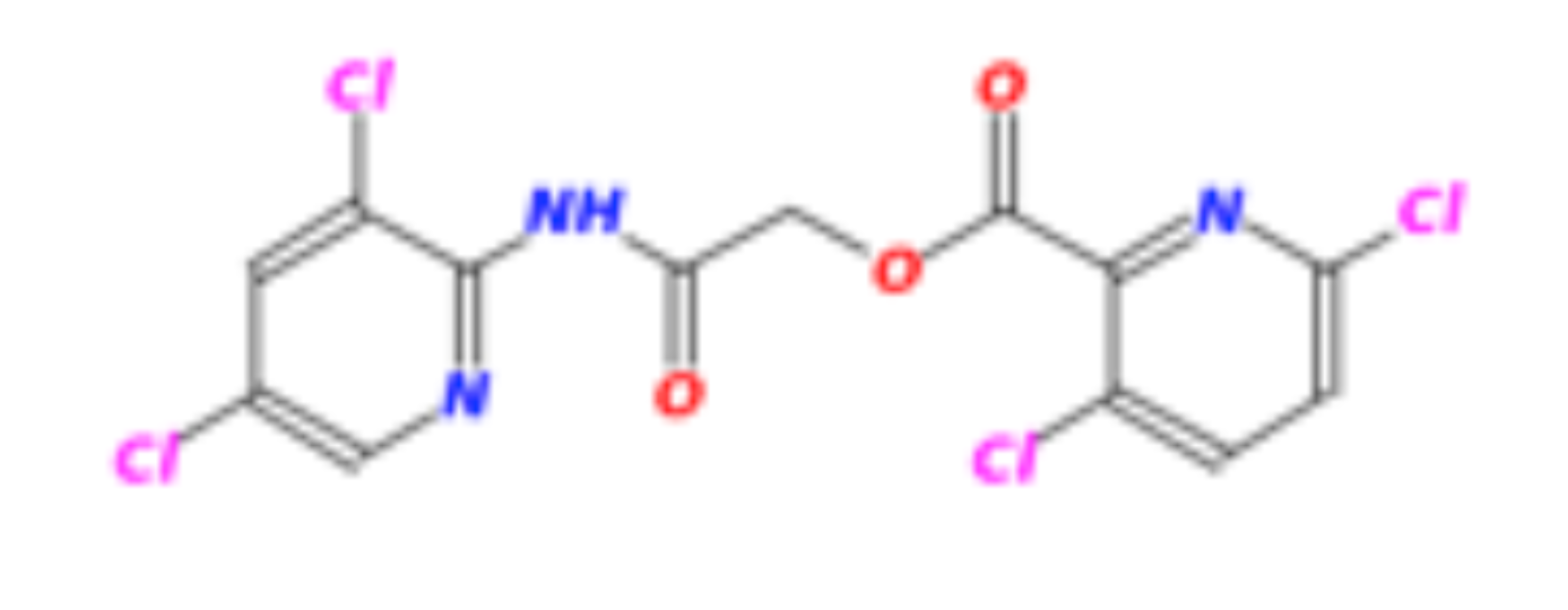 |
| 04767282 | ZINC3 | [(5-chloro-2-pyridyl)carbamoylmethyl](http://zinc.docking.org/synonym/%285-chloro-2-pyridyl%29carbamoylmethyl) | 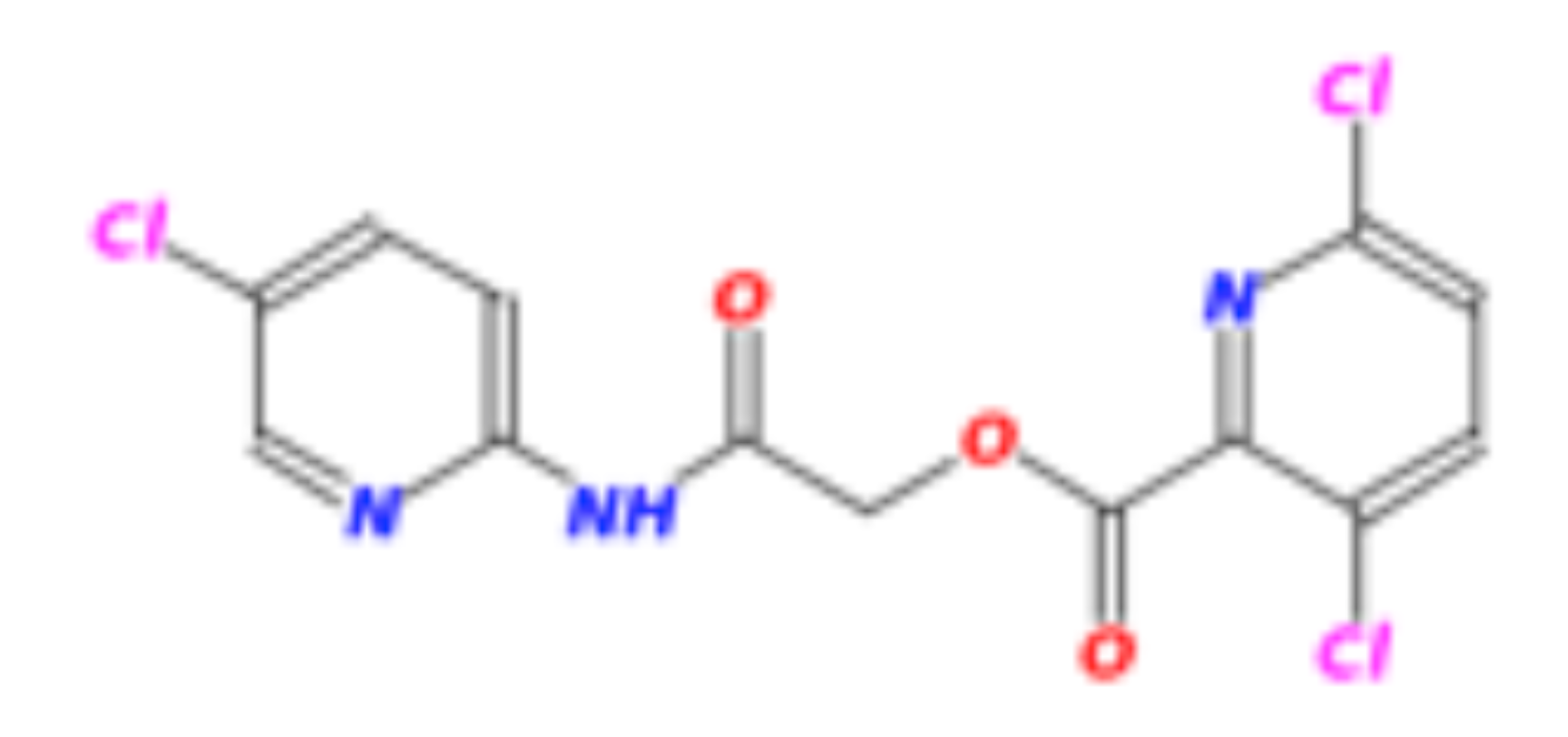 |
| 05752323 | ZINC4 | [[3-chloro-5-(trifluoromethyl)-2-pyridyl]carbamoylmethyl](http://zinc.docking.org/synonym/%5B3-chloro-5-%28trifluoromethyl%29-2-pyridyl%5Dcarbamoylmethyl) | 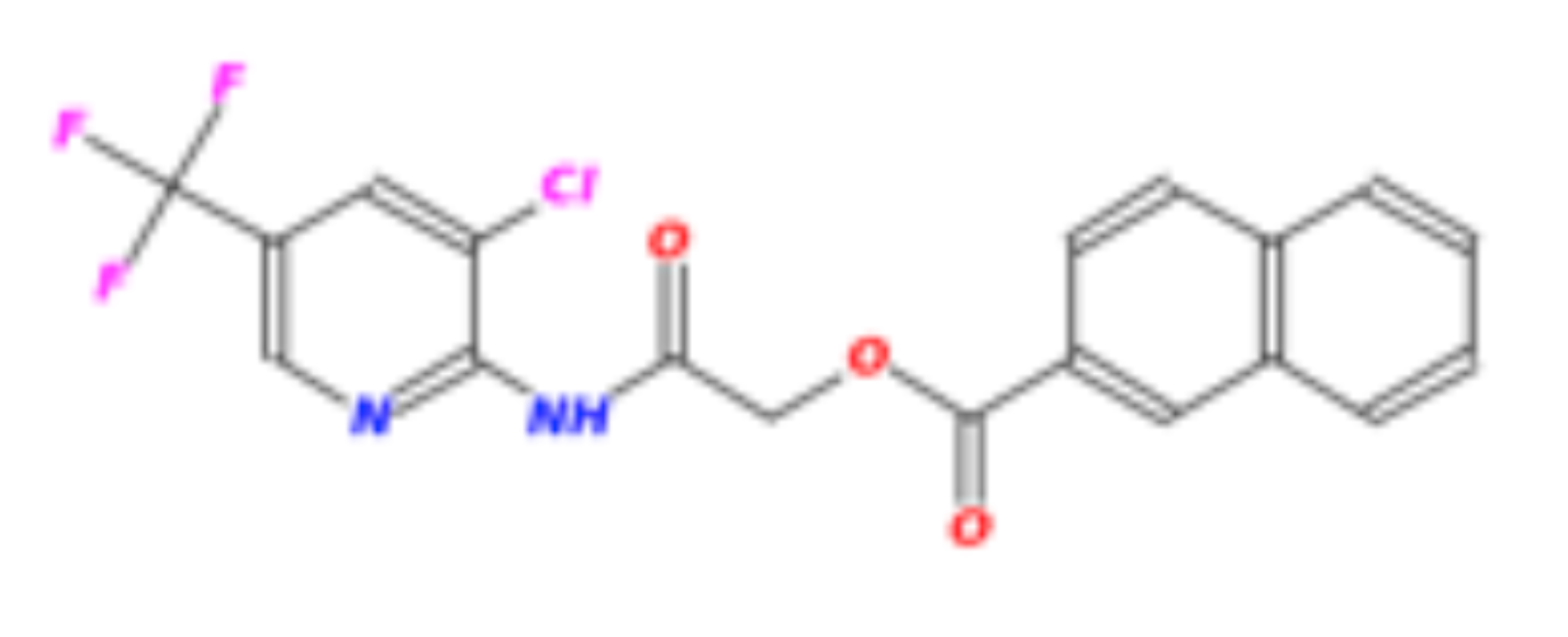 |
| 03369392 | ZINC5 | [2-(2-fluorophenyl)acetic-acid-[2-[(5-chloro-2-pyridyl)amino]-2-keto-ethyl]-ester](http://zinc.docking.org/synonym/2-%282-fluorophenyl%29acetic-acid-%5B2-%5B%285-chloro-2-pyridyl%29amino%5D-2-keto-ethyl%5D-ester) | 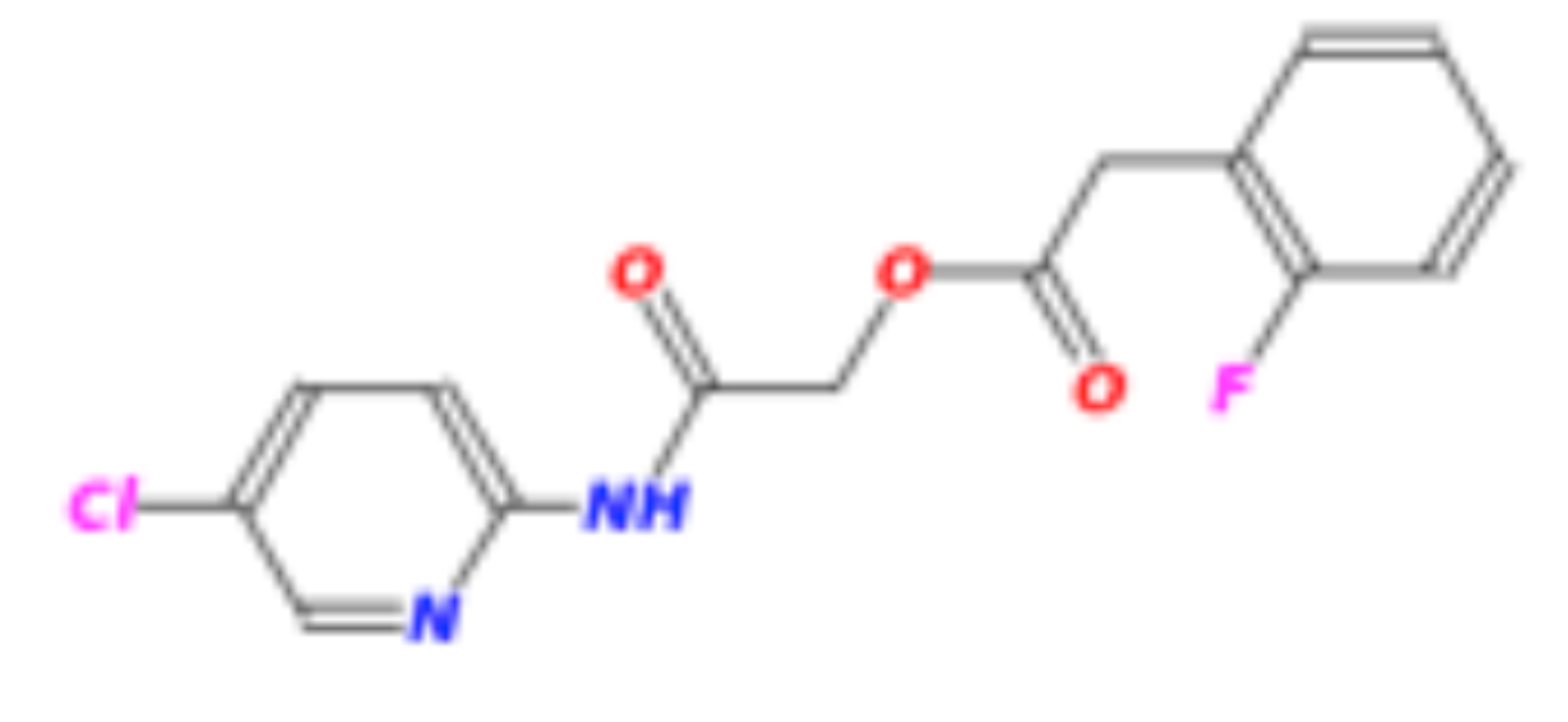 |
| 03270295 | ZINC6 | [2-chloronicotin-[2-[(3,5-dichloro-2-pyridyl)amino]-2-keto-ethyl]-ester](http://zinc.docking.org/synonym/2-chloronicotin-%5B2-%5B%283%2C5-dichloro-2-pyridyl%29amino%5D-2-keto-ethyl%5D-ester) | 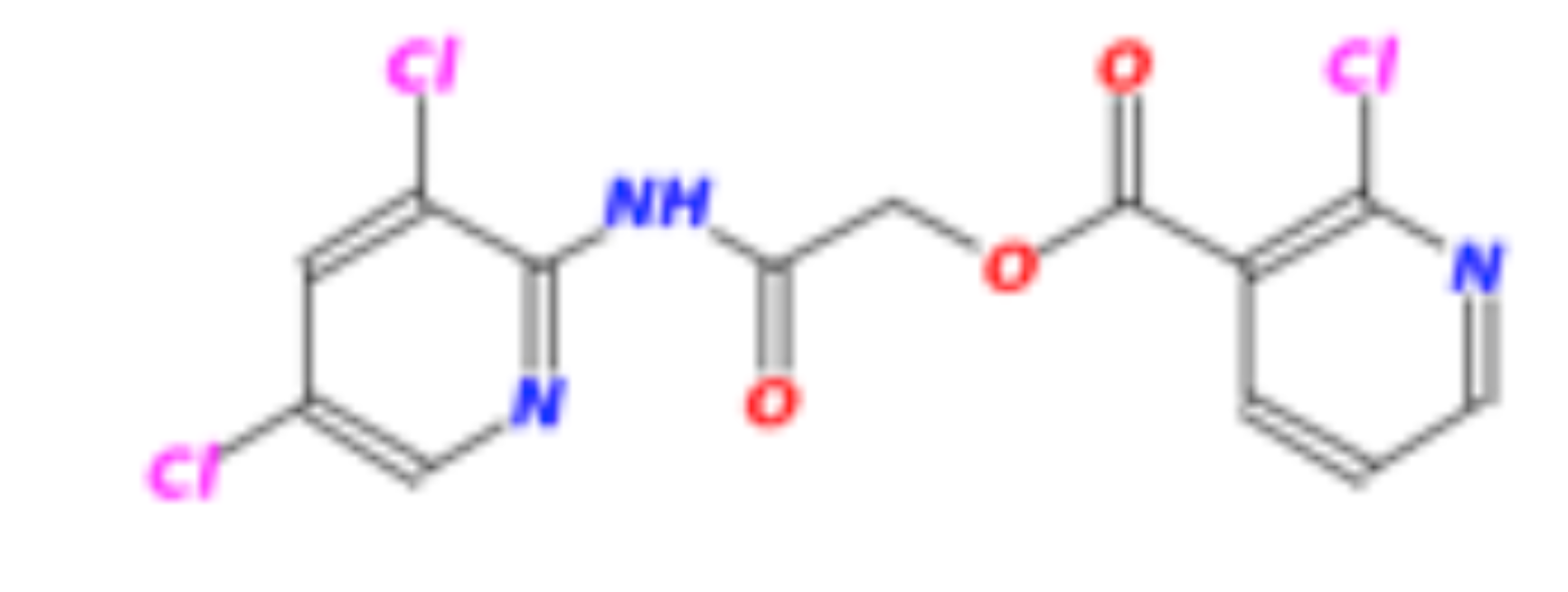 |
| 03269263 | ZINC7 | [anthracene-9-carboxylic-acid-[2-[[3-chloro-5-(trifluoromethyl)-2-pyridyl]amino]-2-keto-ethyl]-ester](http://zinc.docking.org/synonym/anthracene-9-carboxylic-acid-%5B2-%5B%5B3-chloro-5-%28trifluoromethyl%29-2-pyridyl%5Damino%5D-2-keto-ethyl%5D-ester) | 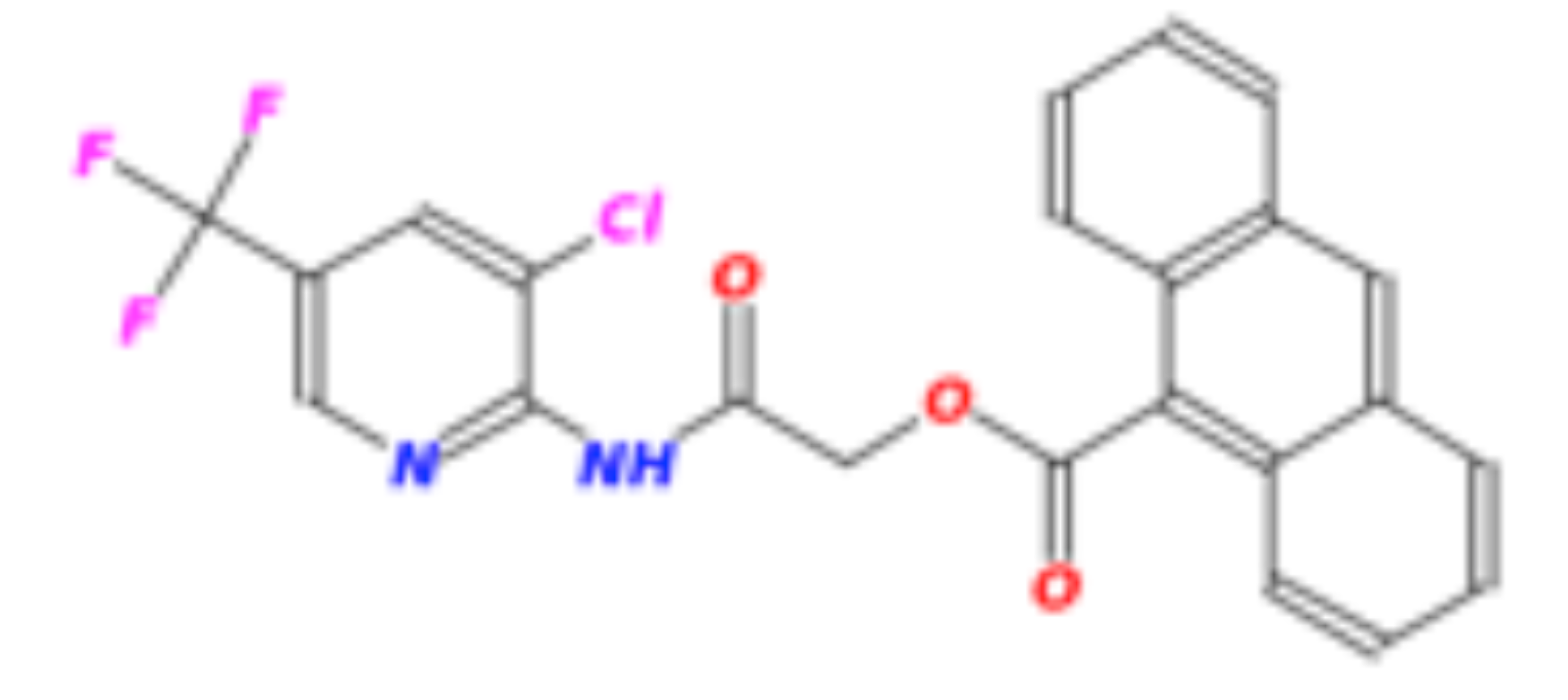 |
| 03269261 | ZINC8 | [naphthalene-1-carboxylic-acid-[2-[[3-chloro-5-(trifluoromethyl)-2-pyridyl]amino]-2-keto-ethyl]-ester](http://zinc.docking.org/synonym/naphthalene-1-carboxylic-acid-%5B2-%5B%5B3-chloro-5-%28trifluoromethyl%29-2-pyridyl%5Damino%5D-2-keto-ethyl%5D-ester) | 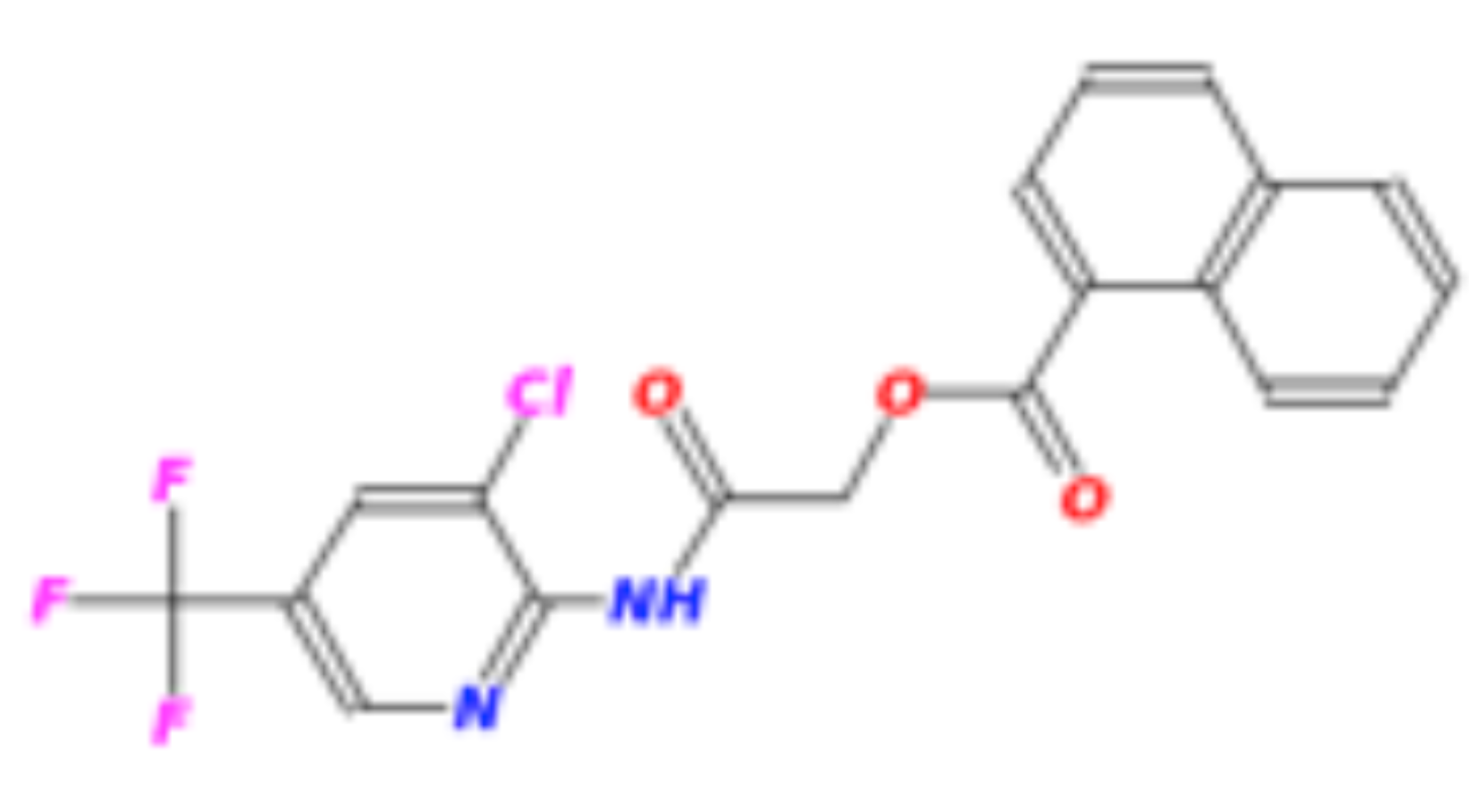 |
| 03264658 | ZINC9 | [2-phenylacetic-acid-[2-[(3,5-dichloro-2-pyridyl)amino]-2-keto-ethyl]-ester](http://zinc.docking.org/synonym/2-phenylacetic-acid-%5B2-%5B%283%2C5-dichloro-2-pyridyl%29amino%5D-2-keto-ethyl%5D-ester) | 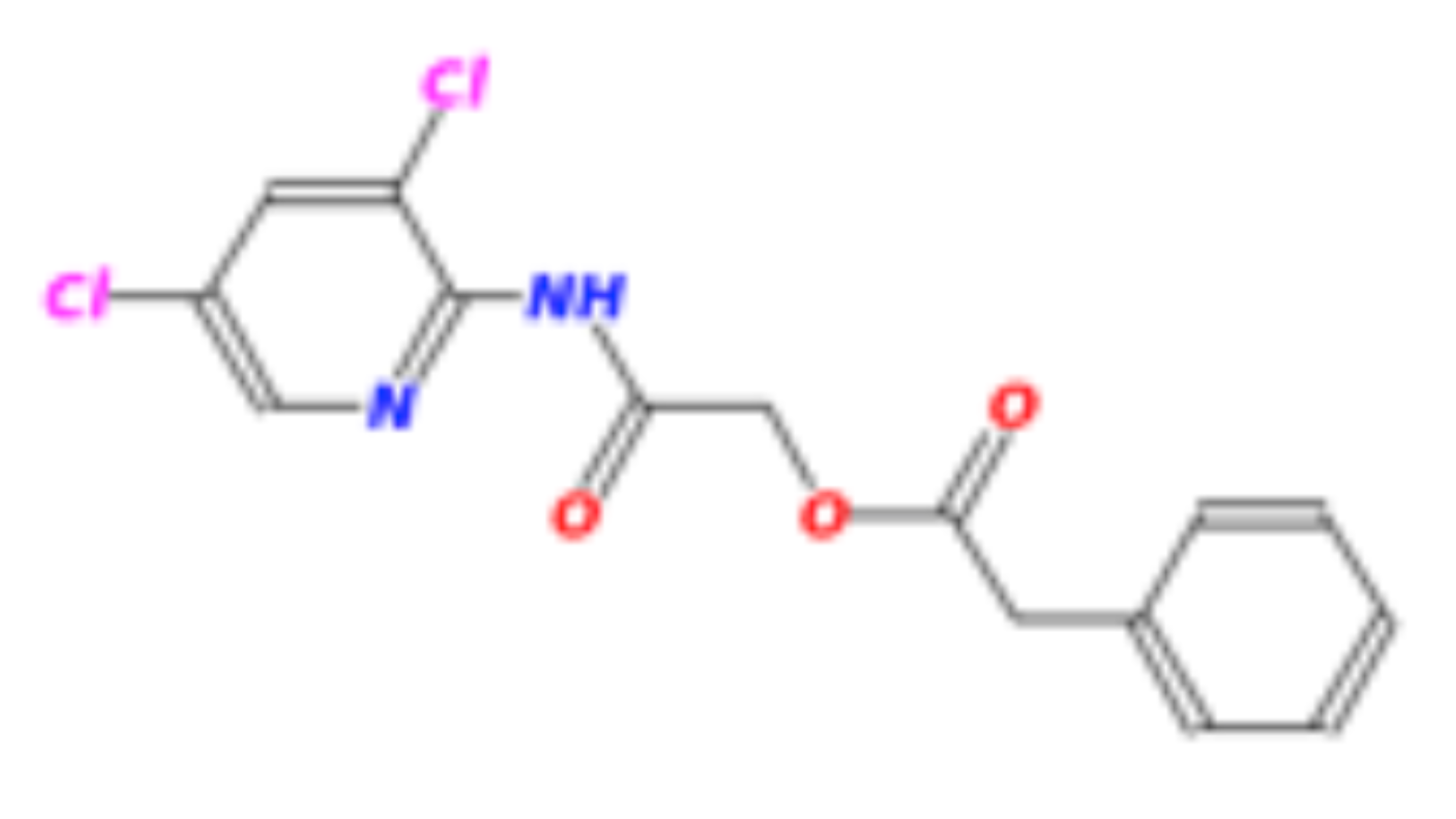 |
| 03260222 | ZINC10 | [picolin-[2-[[3-chloro-5-(trifluoromethyl)-2-pyridyl]amino]-2-keto-ethyl]-ester](http://zinc.docking.org/synonym/picolin-%5B2-%5B%5B3-chloro-5-%28trifluoromethyl%29-2-pyridyl%5Damino%5D-2-keto-ethyl%5D-ester) | 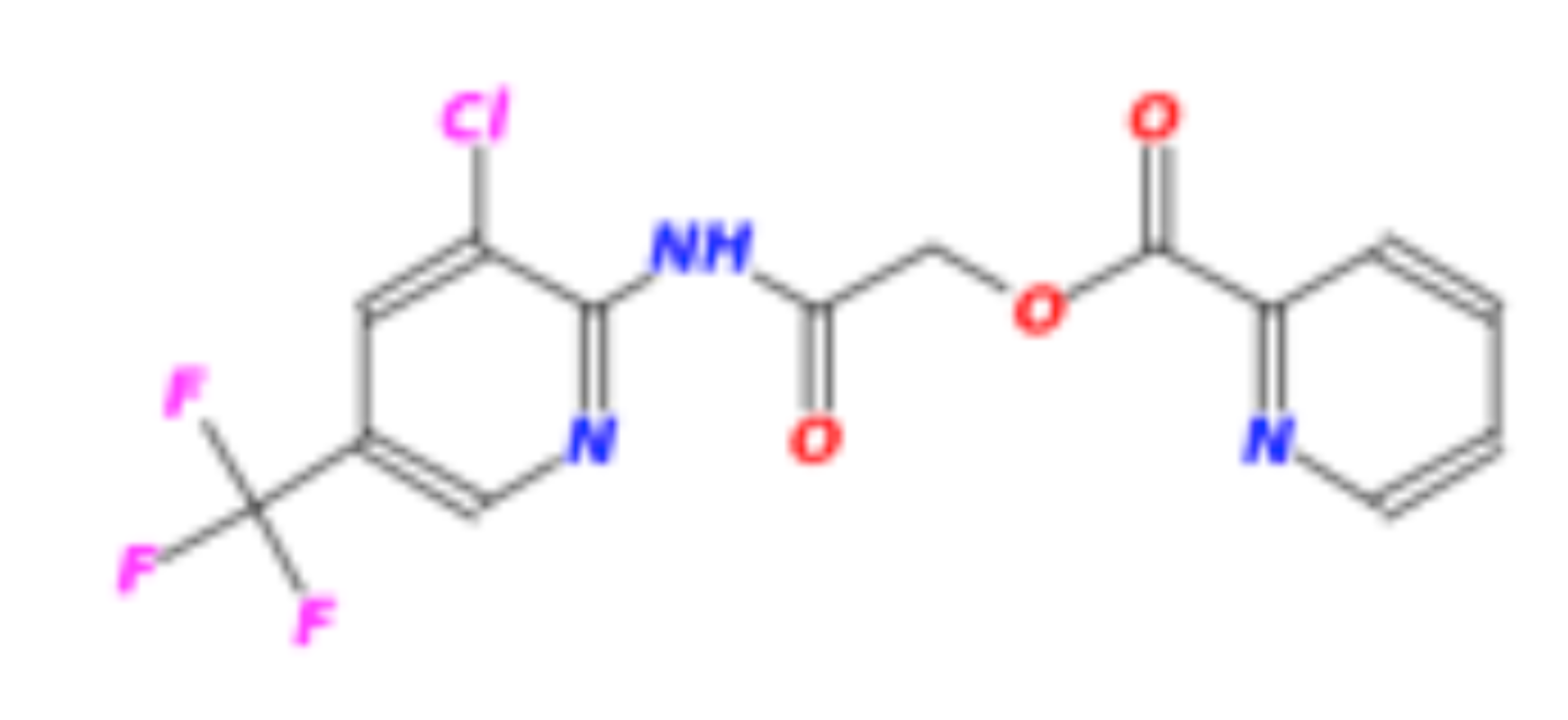 |
| 03257323 | ZINC11 | [6-chloronicotin-[2-[(3,5-dichloro-2-pyridyl)amino]-2-keto-ethyl]-ester](http://zinc.docking.org/synonym/6-chloronicotin-%5B2-%5B%283%2C5-dichloro-2-pyridyl%29amino%5D-2-keto-ethyl%5D-ester) | 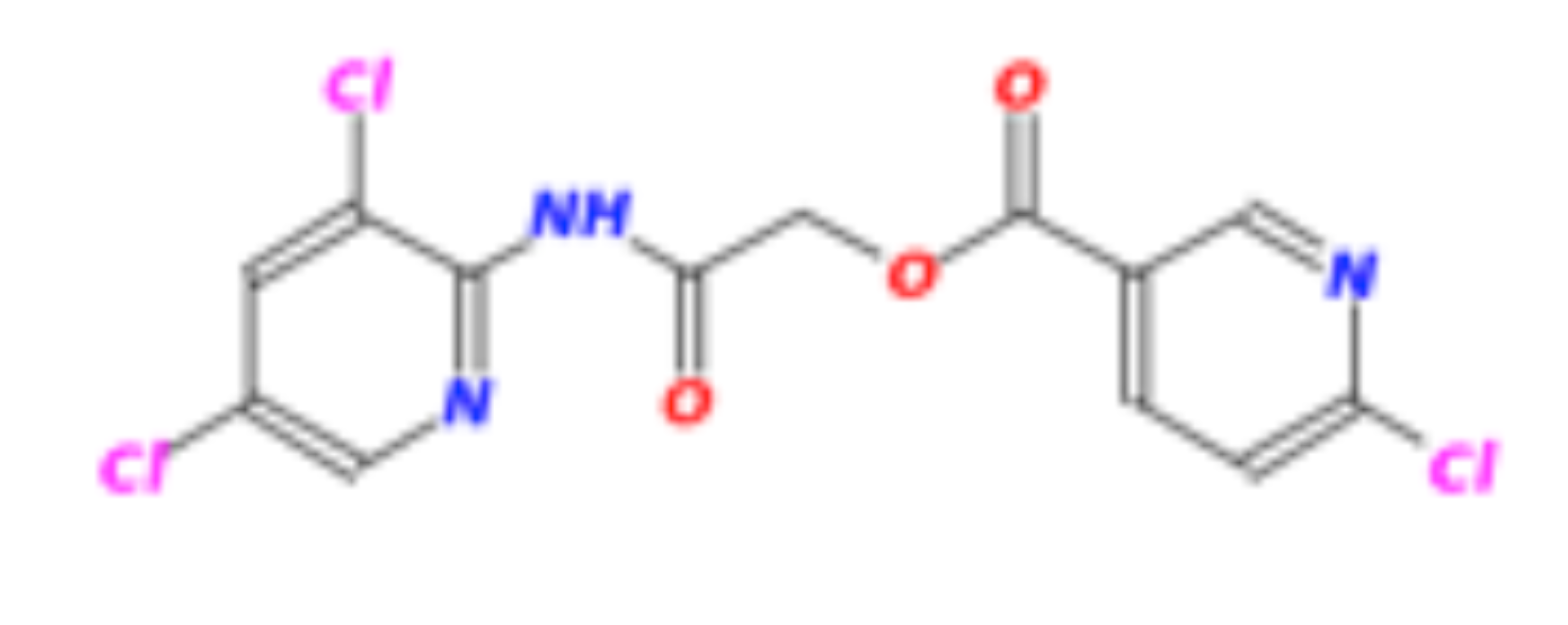 |
| 03434956 | ZINC12 | [2,4-difluorobenzoic-acid-[2-[(5-chloro-2-pyridyl)amino]-2-keto-ethyl]-ester](http://zinc.docking.org/synonym/2%2C4-difluorobenzoic-acid-%5B2-%5B%285-chloro-2-pyridyl%29amino%5D-2-keto-ethyl%5D-ester) | 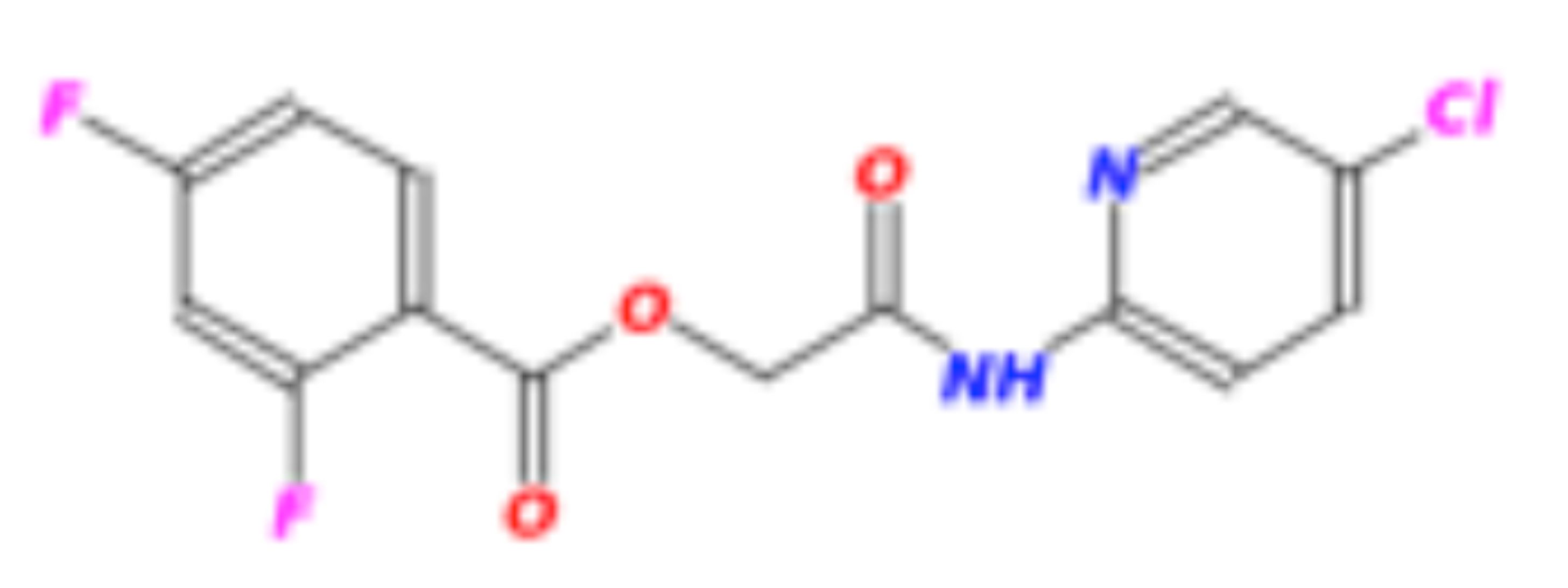 |
| 03425523 | ZINC13 | [2-(2-fluoroanilino)nicotin-(2-amino-2-keto-ethyl)-ester](http://zinc.docking.org/synonym/2-%282-fluoroanilino%29nicotin-%282-amino-2-keto-ethyl%29-ester) | 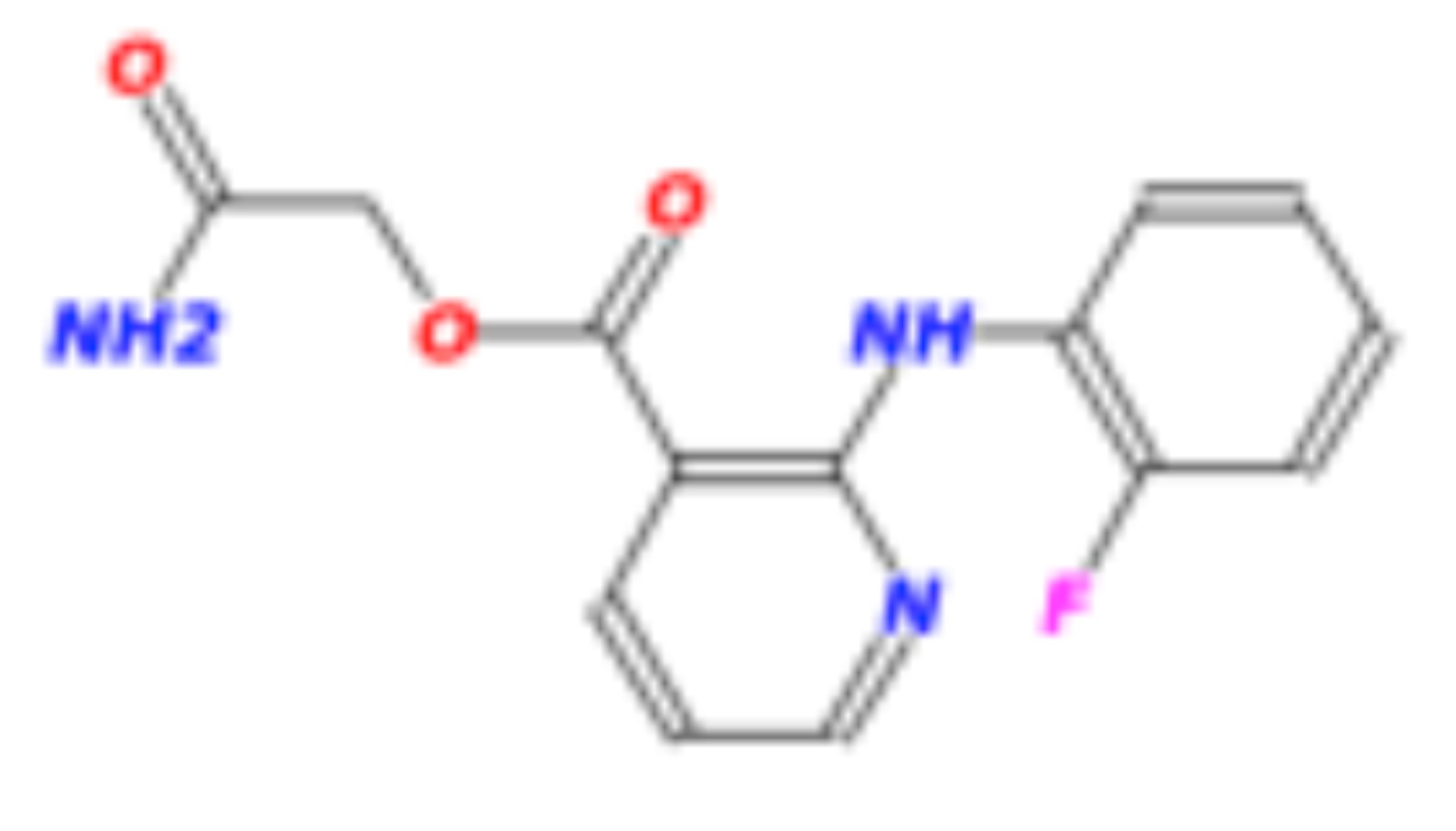 |
| 03401528 | ZINC14 | [3-fluorobenzoic-acid-[2-[(5-chloro-2-pyridyl)amino]-2-keto-ethyl]-ester](http://zinc.docking.org/synonym/3-fluorobenzoic-acid-%5B2-%5B%285-chloro-2-pyridyl%29amino%5D-2-keto-ethyl%5D-ester) | 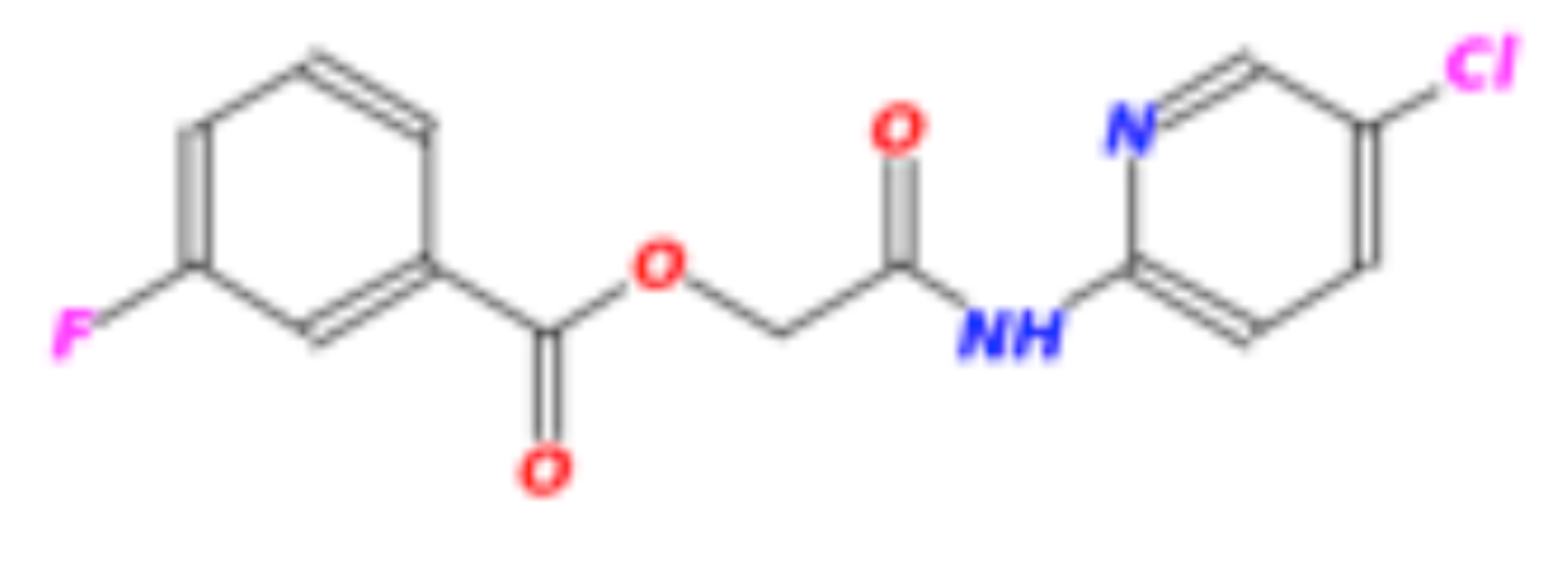 |
| 03379044 | ZINC15 | [4-fluorobenzoic-acid-[2-[(5-chloro-2-pyridyl)amino]-2-keto-ethyl]-ester](http://zinc.docking.org/synonym/4-fluorobenzoic-acid-%5B2-%5B%285-chloro-2-pyridyl%29amino%5D-2-keto-ethyl%5D-ester) | 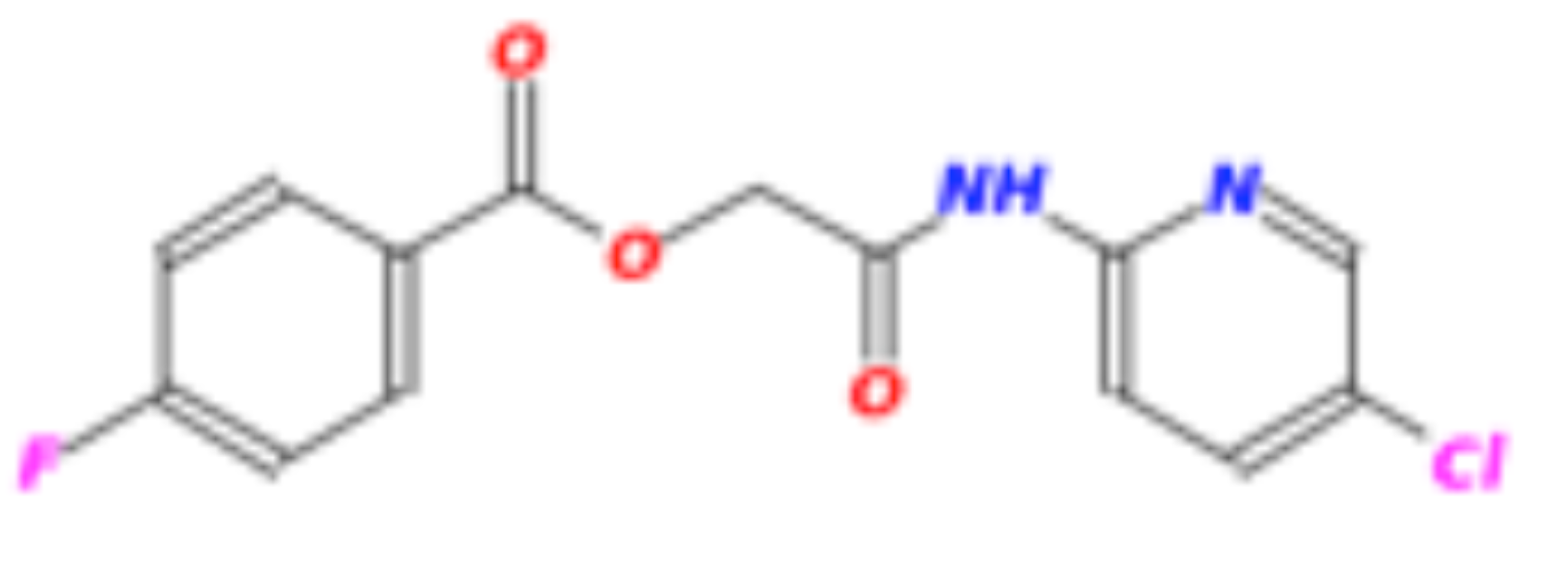 |
